# Benzo[a]pyrene-induced metabolic shift from glycolysis to pentose phosphate pathway in the human bladder cancer cell line RT4

**DOI:** 10.1038/s41598-017-09936-1

**Published:** 2017-08-29

**Authors:** Nisha Verma, Mario Pink, Stefan Boland, Albert W. Rettenmeier, Simone Schmitz-Spanke

**Affiliations:** 1 Institute and Outpatient Clinic of Occupational, Social and Environmental Medicine, University of Erlangen-Nuremberg, Erlangen, Germany; 2 Institute of Hygiene and Occupational Medicine, University Hospital Essen, Essen, Germany; 3 Institute of Medicinal Informatics, Biometry and Epidemiology, University Hospital Essen, Essen, Germany

## Abstract

Benzo[a]pyrene (B[a]P), a well-known polyaromatic hydrocarbon, is known for its lung carcinogenicity, however, its role in bladder cancer development is still discussed. Comparative two-dimensional blue native SDS-PAGE analysis of protein complexes isolated from subcellular fractions of 0.5 µM B[a]P-exposed cells indicated a differential regulation of proteins involved in carbohydrate, fatty acid, and nucleotide metabolism, suggesting a possible metabolic flux redistribution. It appeared that B[a]P exposure led to a repression of enzymes (fructose-bisphosphate aldolase A, glucose-6-phosphate isomerase, lactate dehydrogenase) involved in glycolysis, and an up-regulation of proteins (glucose-6-phosphate 1-dehydrogenase, 6-phosphogluconolactonase) catalyzing the pentose phosphate pathway and one carbon metabolism (10-formyltetrahydrofolate dehydrogenase, bifunctional purine biosynthesis protein). Untargeted metabolomics further supported the proteomic data, ﻿a lower concentration of glycolytic metabolite was observed as compared to glutamine, xylulose and fatty acids. The analysis of the glutathione and NADPH/NADP^+^ content of the cells revealed a significant increase of these cofactors. Concomitantly, we did not observe any detectable increase in the production of ROS. With the present work, we shed light on an early phase of the metabolic stress response in which the urothelial cells are capable of counteracting oxidative stress by redirecting the metabolic flux from glycolysis to pentose phosphate pathway.

## Introduction

Benzo[a]pyrene (B[a]P) is a widely distributed environmental contaminant. B[a]P, which like other polycyclic aromatic hydrocarbons, is produced as a result of incomplete combustion of organic matter, fossil fuel, tar deposits and charbroiled food. It is also one of the most studied components of cigarette smoke and is known for its mutagenic and carcinogenic properties^1^. Its role in lung, breast, prostate, cervical, and liver carcinogenesis is known^2–4^, however, epidemiological analyses have not yet clearly documented B[a]P or any other polycyclic aromatic hydrocarbon (PAH) as significant candidates for initiating bladder cancer development^5^. Therefore, the risk that exposure to any of these compounds causes bladder cancer is still discussed^6, 7^.

In our previous studies designed to shed some light on the role of B[a]P in bladder cancer development, we found a strong B[a]P - uptake by urothelial cells, the conversion of B[a]P to the oxidative metabolite 3-OH-B[a]P^8^, and an enhanced expression of proteins involved in DNA repair and the intrinsic mitochondrial apoptotic pathway^9^. In the present investigations, we extended this precedent work by analyzing the shift in cellular metabolism that the cells undergo to sustain the hostile environment generated by B[a]P-induced toxicity. For this purpose we applied the two-dimensional blue native SDS-PAGE (2D BN/SDS-PAGE) technique to elucidate the network of protein-protein interactions that regulate cellular metabolism and hence the toxicity of B[a]P. This technique enables isolation of native protein complexes according to their molecular weight (1^st^ dimension) before they are separated into individual proteins (2^nd^ dimension), hence retaining the protein-protein interactions among the multiprotein complexes^10^.

Furthermore, in order to analyze the effects of B[a]P-induced protein alterations at the metabolite level, untargeted metabolomic profiling of B[a]P-exposed cells was carried out by using gas chromatographic mass spectrometric analysis (GC-MS). The untargeted acquisition was used to gain a holistic view of the changes in metabolites occurring upon B[a]P exposure in bladder epithelial cells. This technique was preferred because of its comprehensive approach of taking into account the information of both anticipated and non-anticipated compounds during identification. This apporach can be more successful in monitoring metabolites that play a key role in specific biochemical processes upon B[a]P exposure, irrespective whether or not they have already been identified^11^.

By combining 2D BN/SDS-PAGE technique and metabolomics, we observed alterations of proteins and metabolites involved in cellular metabolism, particularly of those involved in maintaining the redox homeostasis of the cells. It appeared that by alteration of proteins involved in detoxification, and by redirecting energetic substrates and metabolic intermediates into the biochemical pathways that generate key antioxidant molecules, mainly pentose phosphate pathway (PPP) derived NADPH or glutaminolysis-derived GSH, B[a]P exposed cells can directly promote ROS detoxification mechanism.

## Material and Methods

### Reagents

Standard chemicals for 2D BN/SDS-PAGE and mass spectrometric analyses were purchased from Bio-Rad (Munich, Germany), USB Corporation (Cleveland, OH, USA), Merck (Darmstadt, Germany), and Sigma-Aldrich GmbH (Steinheim, Germany). B[a]P, dimethyl sulfoxide (DMSO), *n*-hexane (HPLC grade), and *n*-pentane (HPLC grade) were obtained from Merck. Fructose-6-phosphate and glucose-6-phosphate dehydrogenase were purchased from Alfa Aesar (Karlsruhe, Germany). 6-Phosphogluconic acid and glucose 6-phosphate were purchased from Sigma-Aldrich GmbH (Steinheim, Germany). NADP^+^ was obtained from Serva (Heidelberg, Germany). The NADP/NADPH Quantitation Kit was purchased from PromoKine GmbH (Heidelberg, Germany).

### Cell culture and B[a]P exposure

The human bladder cancer cell line RT4 (ATCC^®^ HTB-2^™^) was cultured until confluency in McCoy’s 5 A medium, supplemented with 10% fetal bovine serum, 7.4 mg/mL L-glutamine, 100 units/mL penicillin, and 100 mg/mL streptomycin at 37 °C in a humidified atmosphere of 95% air and 5% CO_2_. After two days of culture, cells were exposed to 0.5 µM B[a]P dissolved in DMSO (<0.1% of final volume) for 24 h. The same percentage of DMSO was used to expose control cells in all experiments.

### ROS assay

The production of ROS by RT4 cells was measured by using the fluorescent dye 2′,7′-dichlorodihydrofluorescein diacetate (H_2_DCFDA; Invitrogen, Langenselbold, Germany). For the experiment, 100,000 RT4 cells/well were seeded into 96-well plates. Cells at confluence were exposed to different B[a]P concentrations ranging from 0.5 to 10 µM for 24 h. Cells treated with normal cell culture medium were used as negative controls, while 100 μM of H_2_O_2_ served as positive control. The cells were then washed with PBS and incubated with 10 μM H_2_DCFDA in PBS for 20 min at 37 °C. The fluorescence was measured at an excitation wavelength of 485 nm and an emission wavelength of 535 nm.

### TdT-mediated dUTP-X nicked end labeling (TUNEL) assay

Terminal deoxynucleotidyl transferase-mediated biotinylated UTP nick end labeling (TUNEL) was performed using the *in situ* death detection kit, TMR red (Roche Diagnostics GmbH, Germany) according to manufacturer’s instruction. Briefly, 20,000 cells/chamber were seeded in eight-well chamber slides (BD Falcon, Heidelberg, Germany). After 24 h of culture, the cells were exposed to 0.1 to 100 µM B[a]P for another twenty four hours. Finally, the epithelial cells were fixed with 4% paraformaldehyde in PBS (pH 7.4) and permeabilized with freshly prepared 0.1% Triton X-100 in 0.1% sodium citrate. Cells were subsequently labelled with TUNEL working solution. Apoptotic cells were identified as red fluorescent TUNEL-positive cells by fluorescence microscopy, and were normalized to total number of cells as determined by DAPI nuclear staining.

### BrdU cell proliferation assay

100,000 cells/well were seeded into a 96-well plate and exposed for another 24 h to B[a]P at concentrations ranging from 0.1 to 100 µM. BrdU labeling (100 μM) was performed 3.5 h before the end of the exposure. After exposure, the cells were washed (PBS/10% FCS), fixed in an acidic ethanol solution (70% ethanol, 7.5% HCl 4 M) at −20 °C for 30 min, and then incubated with nuclease at 37 °C for an additional 30 min. The fixed cells were washed three times (PBS/10% FCS) and incubated with anti-BrdU antibodies at 37 °C for 30 min. Excess of antibodies was washed off with 1% aqueous BSA solution, and the cells were incubated with peroxidase substrate containing 0.1% substrate enhancer for 30 min until a color became visible. A microplate reader (absorbance wavelength 405 nm; reference wavelength 490 nm) measured the absorbance. The assay was repeated in four independent experiments (*n* = 4).

### NADP^+^ and NADPH extraction

#### Preparation of whole cell lysate

10^5^ RT4 cells were extracted, washed, and the final pellet was resuspended in 200 μL of ice-cold extraction buffer that was provided with the NADP*/*NADPH Quantitation Kit (Promokine, Heidelberg, Germany). The cells were then subjected to homogenization by using a mixer mill with steel grinding balls (MM200, Retsch, Haan, Germany) for 5 min at maximum frequency. The samples were centrifuged at 13,000 × g for 5 min at 4 °C.

#### Preparation of cytosolic lysate

10^6^ RT4 cells were extracted, washed, and the final pellet was resuspended in 1 mL of ice-cold PBS, followed by centrifugation at 100 × g at 4 °C. The cell pellet was then resuspended in 400 µl of ice-cold cytosolic buffer (150 mM NaCl, 50 mM HEPES pH 7.4, 25 µg/ml digitonin, freshly added protease inhibitors). The suspension thus obtained was further incubated at 4 °C for 10 min followed by centrifugation at 2000 × g to pellet the cells. The supernatant was aspirated and used as cytosol enriched fraction.

#### Analysis NADPt and NADPH content of the cells

The supernatants thus obtained from above two preparations, were again centrifuged and concentrated by using 10 kDa cutoff centrifugal filters units (Millipore, Darmstadt, Germany) at 13,000 × g for 10 min at 4 °C to remove NADPH- or NADP^+^-consuming enzymes. The total NADPH and NADP^+^ pool (NADPt) was determined in the through-flow, and the resulting value was compared to standard curves. To detect NADPH only, aliquots of 200 μL of homogenized samples were heated to 60 °C for 30 min to remove the thermosensitive NADP^+^ from the solution. The samples were then cooled on ice and centrifuged at 13,000 × g for 1 min at 4 °C. Each sample and standard were diluted (as described in the protocol provided with the kit) to make up a 50 μL sample volume. NADP^+^ cycling enzyme mix and enzymatic buffer (100 μL) as provided in the kit were added to the samples and incubated for 5 min at RT. NADPH developer (10 μL) was added, and the sample was incubated for up to 4 h. The absorbance was read at 450 nm.

### Determination of glutathione (GSH) content of the cells

Glutathione was quantified by using a commercially available kit obtained from Sigma Aldrich. For the assay, RT4 cells were extracted, washed, and suspended in PBS to a cell density of 5 × 10^8^ cells per mL in an Eppendorf tube. The cells were centrifuged at 600 × g for 10 min to obtain a packed pellet. The volume of the pellet was weighed and resuspended in 3 volumes of 5% 5-sulfosalicylic acid solution provided with the kit. The cells were then subjected to homogenization by using a mixer mill with steel grinding balls (MM200, Retsch, Haan, Germany) for 5 min at maximum frequency. Following centrifugation of the samples at 10,000 × g for 10 min at 4 °C, the total GSH content was determined in the through-flow and the obtained value was compared to standard curves. 10 µL of each sample and standard were added to a 96-well plate. 150 µL of 1X assay buffer (100 mM PBS (pH 7) with 1 µM EDTA) containing 228 μL of the diluted glutathione reductase and 228 μL of 5,5′-dithiobis(2-nitrobenzoic acid) stock solution provided with the kit was then added to each well and incubated for 5 min at RT. NADPH solution (50 μL) was added, and the absorbance was read at 412 nm.

### Glucose-6-phosphate dehydrogenase (G6PD) activity

15,000 cells/well were seeded into a 96-well plate. After 24 h the cells were exposed to 0.5 µM B[a]P. The plates were washed three times with PBS and kept at −80 °C to lyse the cells. The test was performed using an assay buffer containing 5 mM MgCl_2_, 2.5 mM glucose 6-phosphate, 250 µM of NAPD^+^ (as substrate) and 0.1% triton in PBS. As inhibition control, 100 µM NADPH was added to the desired wells. NADPH production was monitored by reading absorbance at 340 nm in a Tecan plater reader every two minutes. The velocity (340 nm absorbance/time) was calculated and used as enzyme activity. One unit of G6PD enzyme activity is defined as 1 µmol of NADPH released per minute per 10^6^ cells at 35 °C.

### Glucose-6-phosphate isomerase (GPI) activity

For the experiment, 15,000 RT4 cells/well were seeded into 96-well plates. The next day, the cells were exposed to 0.5 µM B[a]P for 24 h. The 96-well plates were washed three times with PBS and kept at −80 °C to facilitate cell lysis. The GPI enzyme assay was performed using reaction mixture containing 100 µM Tris-HCl (pH 7.6), 0.5 mM NADP^+^, 2 mM EDTA, 1 U/well of glucose-6-phosphate dehydrogenase and 0.1% triton. 6-Phosphogluconic acid (5 mM) was used as an inhibitor and the reaction was started by addition of fructose-6-phosphate to a concentration of 1 mM. NADPH formation was measured by reading the absorbance at 340 nm in a plate reader every two minutes. The velocity (340 nm absorbance/time) was calculated and used as enzyme activity. One unit of GPI activity is defined as 1 μmol of glucose 6-phosphate released per minute per 10^6^ cells at 35 °C.

### Two-dimensional blue native/SDS-PAGE (2D BN/SDS-PAGE)

#### Sample preparation and subcellular fractionation of RT4 cells

For the enrichment of organelles, ProteoExtract^®^, a commercially available subcellular fractionation kit (S-PEK, Merck Millipore, Darmstadt, Germany), was applied according to the manufacturer’s instructions. By using the kit, control cells and cells exposed to B[a]P were fractionated into three subcellular compartments: cytosol, membrane/organelles, and nucleus.

In detail, B[a]P-exposed or non-exposed RT4 cells (3.5 × 10^6^) were seeded in 25 cm² culture flasks in 5 mL culture medium. Cells at confluence were harvested, mixed with 1 mL of cold extraction buffer 1 that contained protease inhibitors, and incubated for 10 min at 4 °C (all incubations were performed on an end-over-end shaker). Insoluble material was sedimented at 1000 × g for 10 min at 4 °C, and the resulting supernatant, the cytosolic subproteome, was sampled. The pellet was then mixed with 1 mL of cold extraction buffer 2 and incubated for 30 min at 4 °C. Following separation of the insoluble material by centrifugation at 6000 × g for 10 min at 4 °C, the supernatant membrane/organelle subproteome was removed, and the pellet was mixed with 500 μL of cold extraction buffer 3 containing 1.5 μL benzoase to digest DNA. After 10 min of incubation, the insoluble material was sedimented at 7000 × g for 10 min at 4 °C, and the supernatant nuclear fraction was sampled. To enrich the subcellular fractions and to avoid a carryover of proteins from one fraction to another, the enrichment step for each fraction was repeated up to three times. Fractions were then aliquoted and stored at −80 °C until further use. Protein quantification was made according to the BCA assay.

### 2D BN/SDS-PAGE

Fifty micrograms of protein sample from each subcellular fraction were mixed with 5 µL of sample buffer (750 mM aminocaproic acid and 5% (w/v) CBB G-250) and centrifuged at 14,000 rpm for 10 min at 4 °C. The supernatant was then separated on a 4–16% Bis-Tris polyacrylamide native gradient gel by using the Invitrogen electrophoresis system. The outer chamber was filled with ice-cold anode buffer (50 mM Bis–Tris, pH 7 0) and the inner chamber with ice-cold blue cathode buffer (15 mM Bis–Tris, pH 7.0, 50 mM tricine, and 0.02% CBB G-250). Electrophoresis was performed at 20 mA, 200 V, and 10 W for approx. 4 h at 4 °C and stopped when the tracking line of the CBB G-250 dye had reached the edge of the gel. The lanes from the first dimension were cut into individual strips, and prior to the separation in the second dimension, the strips were equilibrated in denaturation buffer (1% sodium dodecyl sulfate (SDS) and 1% iodoacetamide (IAA)) for 30 min at room temperature and placed into a 12% Bis–Tris polyacrylamide gel of the same thickness. The second-dimension run was performed at 150 V, 75 mA, and 5 W. At the end of the run, the gel was stained with CBB by applying the protocol developed in our laboratory^12^. Briefly, the staining solution was prepared by mixing 5% aluminum sulfate 14–18 hydrate, 10% ethanol, 0.02% CBB G-250, and 8% phosphoric acid. The gels were stained for 3 h followed by destaining for 30 min in a solution containing 2% phosphoric acid and 10% ethanol. Gels were allowed to destain in water overnight before being ready for further processing.

### Image acquisition and analysis

The stained gels were scanned and analyzed according to the method described in our previous publication^9^. Briefly, spot detection, quantification (rel. % volume), and pattern matching were performed by using the Delta2D v4.0 software (Decodon, Greifswald, Germany). Each spot volume was expressed as relative percentage of the total volume of all spots present in a gel. Significant alteration of control to B[a]P ratio was defined as equal to or more than twofold difference in intensity of spots that were present in all of the four gels of each experiment.

### In-gel enzymatic digestion and MALDI-TOF-MS analysis of proteins

Details of the method used for the enzymatic digestion of gels and their MALDI-TOF-MS analysis can be found in our previous publication^9^. Concisely, protein spots of interest were digested using trypsin solution and the digested peptides were then purified by using C-18 Zip tips according to the manufacturer’s protocol. The C18 tips with the absorbed peptides were eluted with matrix solution (10 mg/mL α-cyano-4-hydroxycinnamic acid (CHCA)) onto a MALDI target and analyzed using a Voyager-DE^TM^ STR MALDI-TOF mass spectrometer (Applied Biosystems (AB), Foster City, CA, USA). Proteins were identified by peptide mass fingerprinting utilizing the Mascot v2.0 software (http://www.matrixscience.com) from Matrix Science (Boston, MA, USA) and as peptide search engines the Mass Spectrometry protein sequence DataBase (MSDB) and the National Center for Biotechnology Information (NCBInr) protein databases. Probability-based molecular weight search (MOWSE) scores were estimated by comparing the search result with an estimated random match population and was reported as −10*LOG_10_(P), where P is the absolute probability. Scores greater than 65 were considered statistically significant (p < 0.05).

### Profiling of intermediates of cellular metabolism

#### Extraction of carbohydrate and lipid metabolites from cell lysates

The intermediates of cellular metabolism were extracted according to a combination of solvent system described by Matyash *et al*.^13^, and the procedure used by Strelkov *et al*.^14^, with some modifications. *n*-Hexane/*tert*-butyl methyl ether (1:1) instead of chloroform was used for the extraction of nonpolar substances. Fatty acids were converted to their methyl esters by mild methylation as described by Ichihara *et al*.^15^.

### GC/MS analysis

The isolated and derivatized metabolites were identified and semi-quantified by GC/MS analysis using an HP 6890/5973 GC/MS system (Agilent Technologies, Waldbronn, Germany). The instrument was equipped with a 30 m × 320 µm (i.d.) Optima 5 column coated with a 5% phenyl/95% methylpolysiloxane cross-linked stationary phase (0.25 μm film thickness; Macherey-Nagel, Düren, Germany). Helium was used as carrier gas (flow rate of 1.5 mL/min). The analytes (1 µL each) were injected in the splitless mode with a solvent cutoff time of 6 min. The injector temperature was maintained at 280 °C. The oven temperature was kept at 70 °C for 1 min and then linearly increased at a rate of 1 °C/min up to 76 °C and from there at a rate of 6 °C/min up to 300 °C, where it was maintained for 5 min. The MS was operated in the electron impact (EI) ionization mode at 70 eV with the quadrupole temperature set at 150 °C and the source temperature at 230 °C. Full scans were acquired by repetitively scanning over the mass range from 50 to 550 Da at a scan rate of 500 msec/scan. A standard mixture of alkanes containing C-11, C-14, C-18, C-21, C-24 and C-28 was injected periodically into the GC/MS system to adjust for shifts in retention time. For the same reason, all cell extracts were measured in the presence of a fixed amount of C-30. The resulting GC/MS spectra were deconvoluted with the AMDIS (Automated Mass Spectral Deconvolution and Identification System) software and analyzed with SpecConnect^16^. The resulting relative abundance matrix was used to calculate the metabolite ratios between the various groups. The peak identification was performed with the GOLM metabolome database (Ver. 20100614)^17^ and mass bank^18^.

### Bioinformatic analysis

Bioinformatics tools such as Search Tool for Interacting Chemicals (STITCH) (http://stitch.embl.de/)^19^ and Metabolite Set Enrichment Analysis (MESA) (http://www.metaboanalyst.ca)^20^ were used to elucidate the biological pathways associated with the individually identified proteins and metabolites.

### Statistical analysis

All experiments were performed at least four times with different RT4 cell lysates. The level of statistical significance relative to control was calculated by using the t-test. A p-value of ≤ 0.05 was considered significant.

## Results

### Effect of B[a]P exposure on ROS production

**Figure 1:**
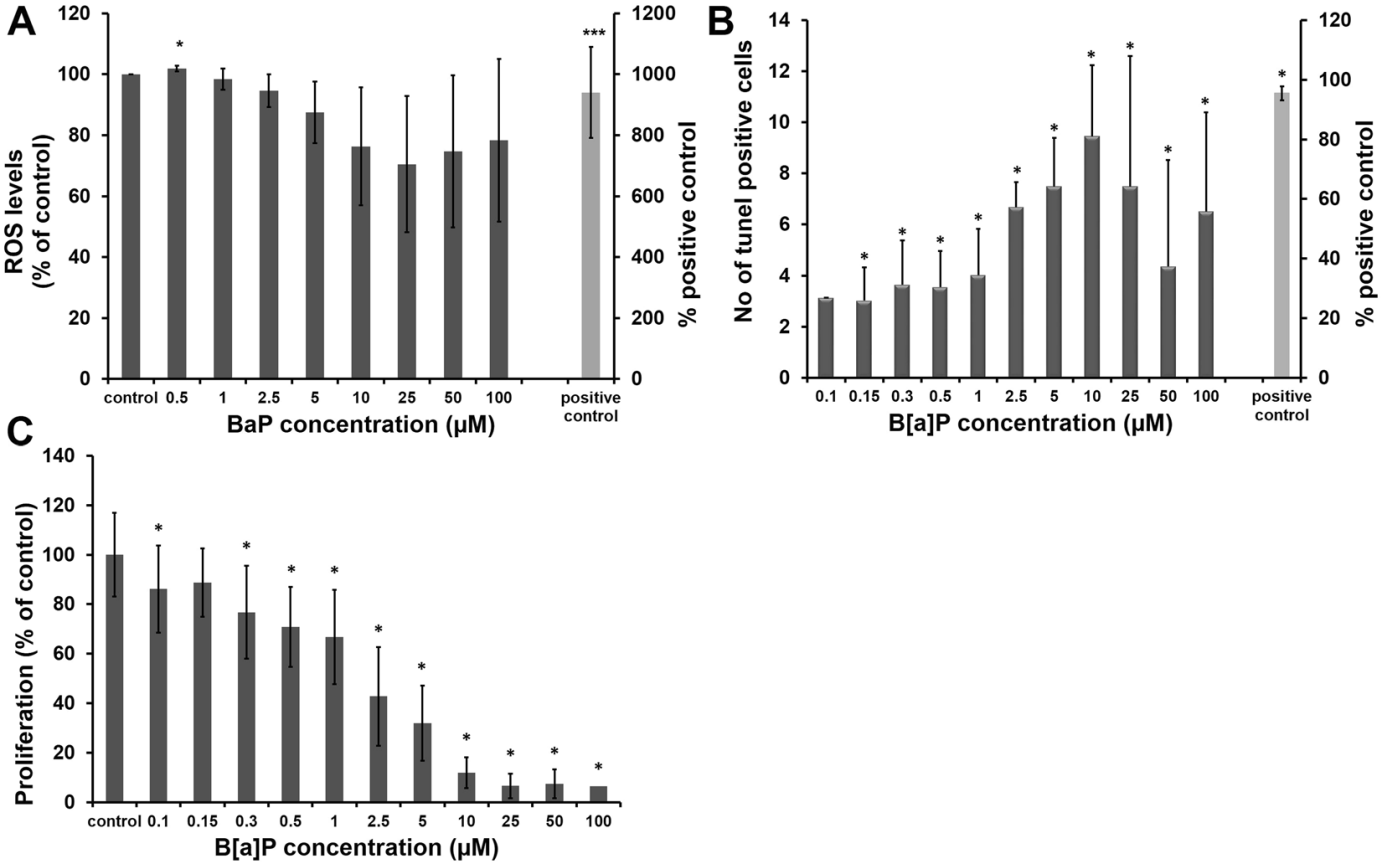
Evaluation of the cytotoxic potential of B[a]P in RT4 cells: To determine the potential of B[a]P to initiate cytotoxic effects in human bladder epithelial cells, various endpoints were measured, including formation of reactive oxygen species (ROS, **A**), apoptosis (TUNEL assay, **B**), cell proliferation (EdU, **C**). Cells were cultured on 96-well plates with a clear bottom and were exposed to different concentrations of B[a]P (0.1 to 100 µM) for 24 h. H_2_O_2_ (100 µM) was used as positive control in ROS assay, while a TUNEL-positive control was obtained by incubation with DNase I. In control, cells were exposed to DMSO (<0.1%). The data is presented as mean ± standard deviation of four independent experiments with RT4 cell lysates. The level of significance relative to the control was determined by using the t-test (*p < 0.05, ***p < 0.001).

B[a]P is a complete carcinogen capable of initiation and promotion. Nevertheless, oxidative stress has been implicated as an important mechanism in B[a]P-induced carcinogenicity. We investigated the potency of B[a]P to induce ROS formation by oxidation of the fluorogenic probe dihydro dichlorofluorescein (H2DCF) to fluorescent DCF in RT4 cells. After 24 h of incubation, only a 3% increase in ROS production was observed for 0.5 µM B[a]P-exposed cells (Fig. 1A), whereas at higher concentrations (5 µM-10 µM B[a]P) no significant increase in ROS production was observed. The cells rather underwent apoptosis as evident from our TUNEL assay results Fig. 1B.

### TUNEL analysis of DNA fragmentation as a biological endpoint of B[a]P toxicity

B[a]P, a chemically unreactive compound, requires metabolic activation by monooxygenases/hydroxylases to induce DNA fragmentation. In order to get some evidence for the formation of reactive B[a]P metabolites under the chosen exposure conditions, the TUNEL assay was applied to assess DNA fragmentation. The percentage of TUNEL-positive cells was determined by counting the number of positively stained and the total number of epithelial cells in 10 randomly selected fields of visions. The number of TUNEL-positive cells increased significantly upon incubation with higher concentrations (i.e. 2.5 µM onwards) of B[a]P, with nine times more apoptotic cells observed for cells exposed to 10 µM B[a]P for 24 h (*n* = 4 independent experiments, Fig. 1B).

### Effect of B[a]P exposure on proliferation of bladder epithelial cells

In order to investigate the cytotoxic effects of B[a]P on RT4 cells, cell proliferation assay was performed. B[a]P inhibited the cell proliferation in a dose-dependent manner for 24 h (Fig. 1C).

### 2D BN/SDS-PAGE analyses of subcellularly fractionated samples of RT4 cells

**Figure 2:**
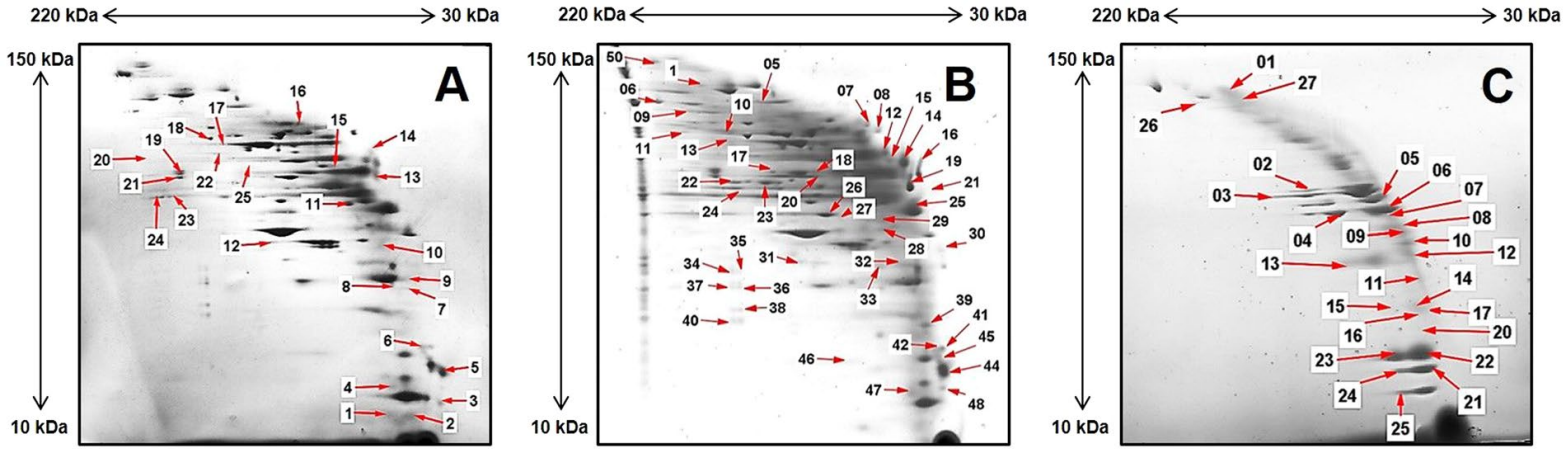
Protein complexes differentially expressed in the subcellular fractions of control and B[a]P-exposed RT4 cells. (**A**) 2D BN/SDS-PAGE gel from the cytosolic, membrane/organelle (**B**), and nuclear fraction (**C**) of RT4 cells exposed to B[a]P (0.5 µM, 24 h). Samples from control and B[a]P-exposed cells were separated and compared by using the Delta2D v4 software. Identified proteins with statistically significant alteration differences between control and exposed cells were marked with arrows. Protein spots of interest were excised, trypsin-digested, and subjected to analysis by MALDI-TOF-MS. The identified proteins are listed with their ID numbers in Suppl. Tables 1, 2 and 3.

The global proteomic response of RT4 cells to B[a]P was profiled by 2D gel electrophoresis (2D BN/SDS-PAGE). Proteins from preparations of five different subcultures from B[a]P-exposed and control cells were subjected to 2D BN/SDS-PAGE. Prior to the 2D BN/SDS-PAGE separation, subcellular fractionation of exposed (0.5 µM B[a]P) and control cells was carried out to concentrate the proteins present in the individual compartments. Over 200 protein spots were quantitatively identified by using the image analysis software Decodon 4.0, among which only those spots were further analyzed which showed a significantly differential alteration of ± 2 between B[a]P-exposed and control cells. Based on this constraint, B[a]P exposure resulted in a differential alteration of 15 proteins in the cytosolic fraction, of 20 proteins in the membrane/organelle fraction, and of 17 proteins in the nuclear fraction. Representative fused 2D BN/SDS-PAGE gel images of all three fractions obtained from control and B[a]P-exposed cells are shown in Fig. 2A–C. Protein spots identified as consistently differentially regulated are marked by numbered arrows. The identity of these proteins was determined by MALDI-TOF-MS (Suppl. Tables 1, 2 and 3).

### Alteration of proteins involved in cellular metabolism

**Figure 3:**
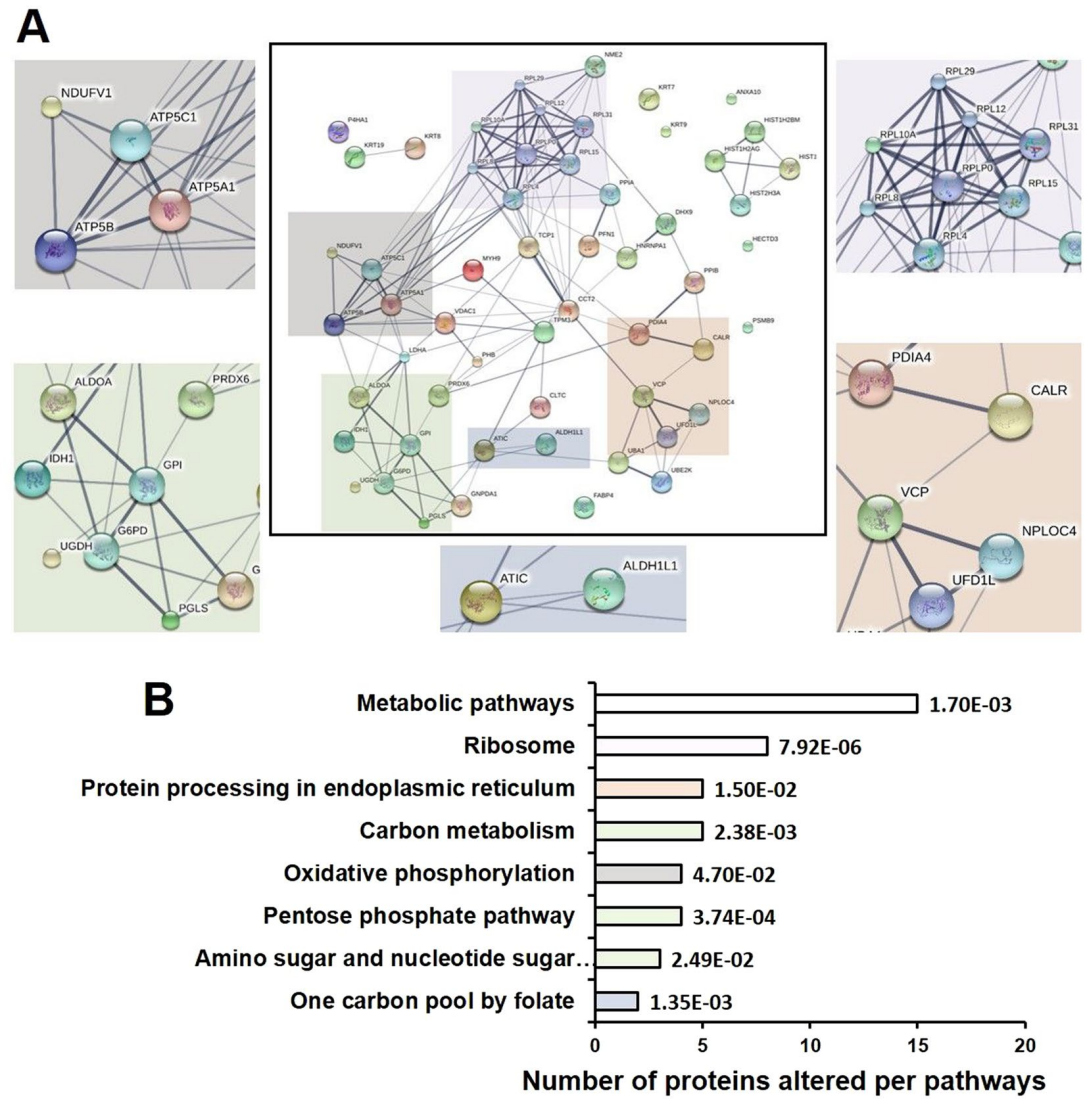
(**A**) Potential protein–protein interactions of all differentially expressed protein species (p < 0.05) associated with B[a]P exposure as suggested by the STITCH database. The analysis was done using the standard settings (medium confidence, network depth 1, no additional white nodes). The color of the connecting lines between two protein species encodes the source of the information: thicker lines signify stronger associations and grey lines represent protein-protein interactions. The pathways of interest are marked in colored boxes. (**B**) KEGG (Kyoto Encyclopedia of Genes and Genomes) Pathway Enrichment Analysis of analyzed proteins. The bar graph represents the number of proteins enriched per pathway and the color represents the corresponding pathways of interest as marked in Fig. 4A (*p < 0.05, FDR (false discovery rate) corrected).

MALDI-TOF-MS analysis revealed that the differentially expressed proteins were involved in cellular functions such as metabolism, antioxidant activity, nucleotide and protein biosynthesis (Suppl. Tables 1, 2 and 3). The data indicated a significant decrease in glycolytic enzymes, such as fructose 1,6-bisphosphate aldolase (ALDA), glucose-6-phosphate isomerase (GPI), and lactate dehydrogenase A (LDHA), however, an up-regulation of glucose-6-phosphate dehydrogenase (G6PD) and 6-phosphogluconolactonase (6PGL), proteins involved in the pentose phosphate pathway (Table 1). Other than the up-regulation of the pentose phosphate pathway, an increased alteration of two proteins involved in one carbon metabolism (bifunctional purine biosynthesis protein PURH, ATIC) and 10-formyltetrahydrofolate dehydrogenase (ALDH1L1) was also observed (Suppl. Table 1). Following MALDI-TOF-MS analysis, functional annotation of the identified proteins was carried out by using the search tool STITCH. This database gives detailed information about the interaction of proteins with small molecules hence giving a comprehensive overview about the involved pathways. Following MALDI-TOF-MS analysis, the proteins were searched for their corresponding official gene name in the Universal Protein Resource (Uniprot) database. The gene counter parts were then uploaded to the STITCH classification system software, which grouped the identified proteins into its respective protein pathways (Fig. 3A,B). The analysis revealed a significant number of proteins (27%) assigned to metabolic pathways, along with pathways involved in carbon metabolism, pentose phosphate pathway, one carbon metabolism and pathways related to amino sugar and nucleotide sugar metabolism. The most interesting observation was the regulation of pathways involved in generation of antioxidant molecules such as pentose phosphate pathway. It appeared that B[a]P exposure lead to alterations of metabolic pathways involved in synthesis of essential building blocks (e.g. proteins and nucleotides) along with the metabolic routes involved in generation of antioxidant molecules. To study whether B[a]P exposure leads to changes in the redox state of the cells as pointed by proteomic studies, enzyme activity of G6PD and GPI along with NADP^+^/NADPH and GSH content of the RT4 cells was assessed.

**Table 1:** List of altered glycolytic and pentose phosphate pathway-associated proteins (≥2) after B[a]P exposure (0.5 µM, 24 h) compared to controls.

^1^Spot ID no	^2^Gene name	^2^Protein name	Subcellular fraction	^3^Regulation
Identified Glycolytic proteins differentially regulated in control and B[a]P-exposed RT4 cells. Reprsesentative images of 2D BN/SDS-PAGE marked with differentially expressed proteins are shown in Fig. 2A.				
ID 24295	ALDA	fructose 1,6-bisphosphate aldolase	Cytosol	−1.82195
ID24958	GPI	glucose-6-phosphate isomerase	Cytosol	−3.04320
ID25983	LDHA	lactate dehydrogenase A	Cytosol	−1.22291
**Identified proteins of pentose phosphate pathway differentially regulated in control and B[a]P -exposed RT4 cells. Representative images of 2D BN/SDS-PAGE marked with differentially expressed proteins are shown in** Fig. 2A.				
ID22471	G6PD	Glucose-6-phosphate dehydrogenase	Cytosol	1.88427
ID22515	6PG	6-phosphogluconolactonase	Cytosol	2.29904

^1^Experimental ID number.

^2^Gene name and protein name entries in Uniprot database.

^3^The values represent the ratio of the relative spot volume of treated and control cells as determined by using the Delta2D v4.0 software.

### Alterations in G6PD and GPI enzyme activity upon B[a]P exposure

**Figure 4:**
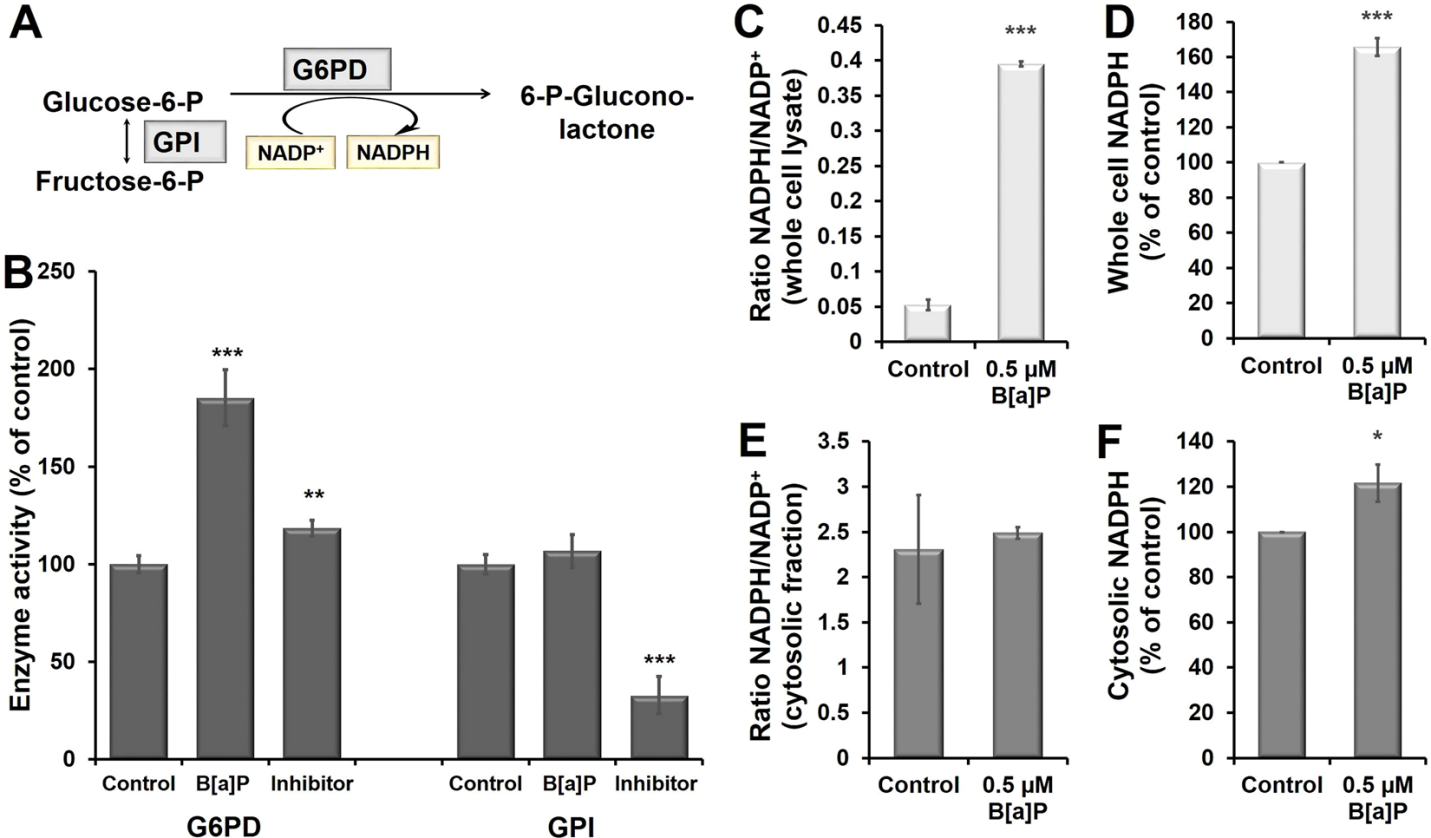
Estimation of cellular cofactors (NADPH and NADPt) and the involved enzymes glucose-6-phosphate dehydrogenase (G6PD) and glucose-6-phosphat-isomerase (GPI). (**A**) Schematic representation of enzymes involved in redirection of the flux from glycolysis to Pentose Phosphate Pathway (PPP). The rerouting of glucose into oxidative PPP depends on a rapid increase of the G6PD flux, which can generate strong inhibitor of GPI thus facilitating a forward flux into PPP. (**B**) Analysis of G6PD and GPI enzyme activity: The enzyme assay was carried out in a 96-well plate in cells exposed to B[a]P (0.5 µM, 24 h). NADPH (100 µM) was used as an inhibitor for G6PD, while 6-PG (5 mM) was used as an inhibitor for GPI enzyme assay. Effects of 0.5 µM B[a]P are shown on cellular (**C**,**D**) and cytosolic (**E**,**F**) NADPH/NADP^+^ ratio and NADPH content of RT4 cells. The data is presented as mean ± standard error of the mean of four independent experiments. The level of significance relative to the control was determined by using the Anova test (*p < 0.05, ***p < 0.001).

During proteomic analysis changes in two of the important glycolytic (G6PD and GPI) enzymes was observed (Fig. 4A). As it is well known, the rerouting of glucose into oxidative pentose phosphate pathway depends on a rapid increase of the G6PD flux, which can generate the strong inhibitor of GPI thus facilitating a forward flux into PPP, we analyzed the enzyme activity of these two enzymes. In B[a]P-exposed cells there was a 85% increase in enzyme activity of G6PD (1.89 U/min/10^6^ cells) as compared to the control (1.02 U/min/10^6^ cells), whereas no significant changes in the GPI enzyme activity was observed in B[a]P exposed cells (4.48 U/min/10^6^ cells) as compared to control (4.21 U/min/10^6^ cells) (Fig. 4B).

### Changes in NADPH/NADP^+^ and GSH content upon B[a]P exposure

The indications of alterations in the pentose phosphate pathway were further confirmed by the measurement of the redox agents NAPDH and NADP^+^ in control and exposed cells. The analysis revealed a 66% increase of NADPH in whole cell lysate while 21% in the cytosolic fraction (Fig. 4D,F). The changes in cellular NADPH content was additionally supported by measurement of corresponding NADPH/NADP^+^ ratios which changed in whole cell samples from 0.052 ± 0.008 in control to 0.395 ± 0.004 in B[a]P exposed cells and from 2.307 ± 0.600 to 2.489 ± 0.063 in cytosolic samples, respectively (n = 4, independent experiments, Fig. 4C,E).

Other than changes in NADPH content, RT4 cells exposed to 0.5 µM B[a]P also revealed a 20% increase in GSH content as compared to the control. By using a standard curve provided with the kit, the intracellular content of control cells and cells exposed to B[a]P amounted to 0.47 µM and 0.56 µM, respectively (Suppl. Fig. 1).

### Profiles of energy metabolites

**Figure 5:**
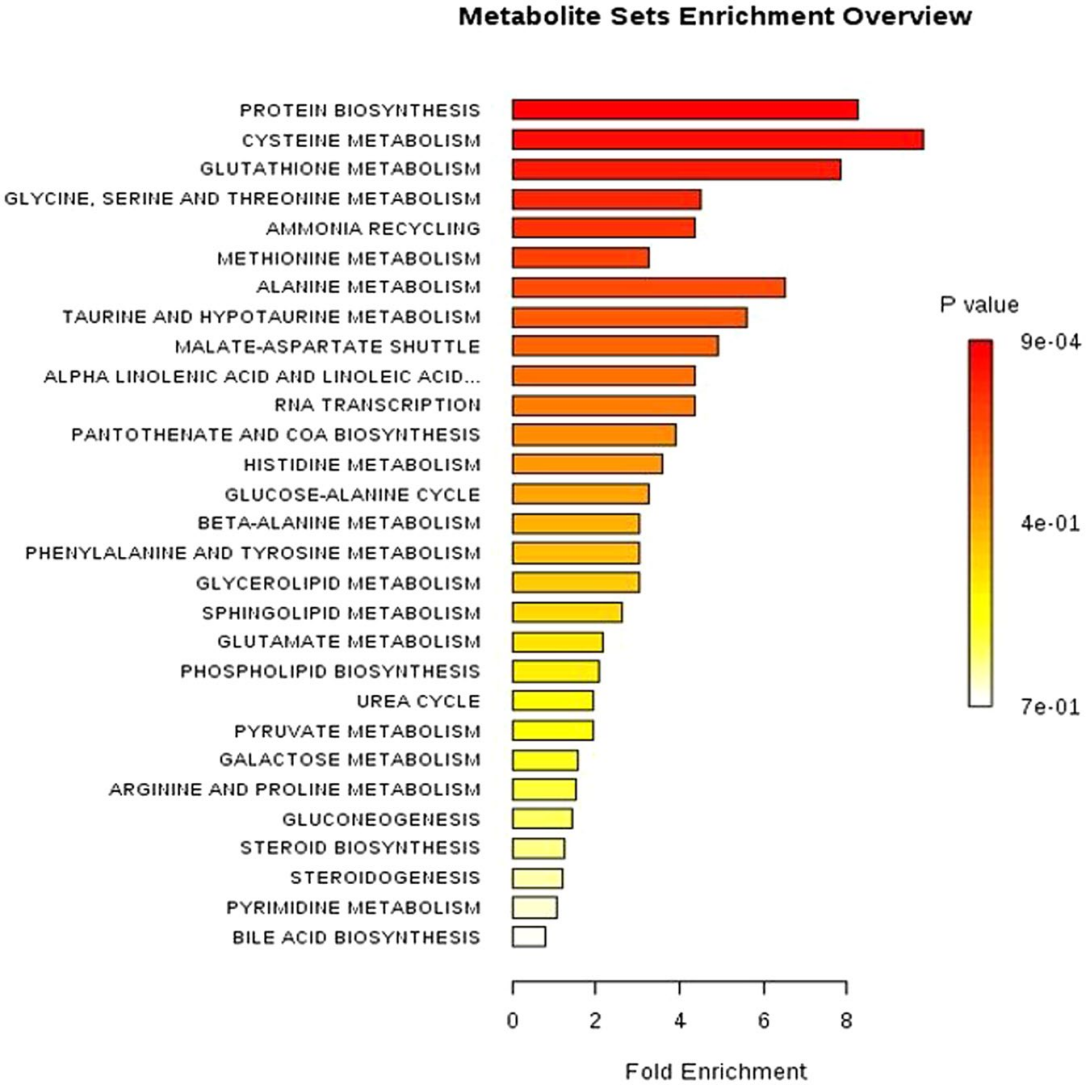
Metabolite Set Enrichment Analysis (MESA) showing biological pathways likely to be active in bladder epithelial cells. The significance of the association between the metabolite list and a certain pathway is expressed by a *ratio* expressing the number of metabolite entries per pathway divided by the total number of metabolites constituting the pathway.

Finally, a metabolomic profile of the B[a]P-exposed cells was established to find out if the observed proteomic changes were reflected in respective alterations at the metabolite level. GC/MS analysis of intermediates of cell metabolism yielded 27 compounds presented in altered concentrations in the B[a]P-exposed cells when compared to control cells. The concentration of a metabolite was considered altered when the relative abundance has changed at least by a factor of ± 1.5. In summary, some fatty acids (oleic acid, gamma-linoleic acid, linolenic acid, stearic acid), amino acids (serine, threonine, phenylalanine, trans-4-hydroxy-L-proline, norleucine, cysteine), and a nucleobase (uracil) were up-regulated, while substrates of carbohydrate metabolism (glycolic acid, lactic acid) and glycerol were present in lower concentrations (Tables 2 and 3). The effects of B[a]P-induced alterations of proteins were also reflected at the metabolite level. In order to determine the biological pathways that might be active in bladder epithelial cells, the sublist of identified metabolites was analyzed using MESA or Metabolite Set Enrichment Analysis (Fig. 5). The method use Over Representation Analysis (ORA) for the enrichment analysis. ORA is implemented using the hypergeometric test to evaluate whether a particular metabolite set is represented more than expected by chance within the given compound list. The significance of the association between the metabolite list and a certain pathway is indicated by a ratio expressing the number of metabolite entries per the pathway divided by the total number of metabolites constituting the pathway; and a p-value (p-values < 0.05 were considered significant). The database highlighted glutathione metabolism, amino acid metabolism, pyrimidine metabolism as one of the many pathways regulated upon B[a]P exposure, thus further validate the results obtained from proteomic analysis.

**Table 2:** Significantly up- or down-regulated metabolites extracted from the RT4 cells with methanol (n = 8) after 24 h exposure of the cells to 0.5 µM B[a]P.

Retention time	Ratio 0.5 µM B[a]P/Control	Purity	Metabolite	ChEBI^1^	Chemical class
6.29 min	−2.43	67%	β-hydroxybutyric acid	20067	ketone bodies
13.71 min	−2.10	90%	glycolic acid	17497	α-hydroxy acid
22.40 min	−1.85	90%	ethanolamine	16000	amino alcohol
22.66 min	1.52	96%	norleucin	18347	amino acid
24.39 min	2.44	92%	uracil	17568	nucleobase
25.22 min	1.60	93%	serine	17115	amino acid
25.89 min	1.77	90%	threonine	16857	amino acid
28.93 min	1.67	85%	trans-4-hydroxy-L-proline	18095	non-proteinogenic amino acid
30.77 min	2.63	88%	phenylalanine	17295	amino acid
30.80 min	1.75	90%	glutamic acid	16015	proteinogenic amino acid
51.91 min	1.50	80%	cholesterol	1307929	steroid

^1^Annotation of the altered metabolites according to the ChEBI database and ontology.

**Table 3:** Significantly up- or down-regulated metabolites extracted from the RT4 cells with *n*-hexane/methyl *tert*-butyl ether (n = 8) after 24 h exposure of the cells to 0.5 µM B[a]P.

Retention time	Ratio 0.5 µM B[a]P/Control	Purity	Metabolite	ChEBI^1^	Chemical class
12.85 min	−1.84	93%	lactic acid	422	carboxylic acid
22.77 min	5.00	91%	pyrophosphoric acid	29888	inorganic compound
22.96 min	−1.57	95%	glycerol	17522	alcohol
32.40 min	1.76	68%	nonadecanoic acid	39246	fatty acid
39.44 min	2.03	53%	sebacic acid	17351	fatty acid
40.82 min	2.14	81%	stearic acid	28842	fatty acid
40.95 min	1.82	77%	γ-linolenic acid	28661	fatty acid
41.16 min	1.53	54%	oleic acid	16196	fatty acid
44.29 min	1.79	57%	D-xylulose	17140	sugar
46.85 min	3.82	33%	cysteine	17561	amino acid
47.93 min	2.09	55%	ciliatine	15573	organic compound

^1^Annotation of the altered metabolites according to the ChEBI database and ontology.

## Discussion

**Figure 6:**
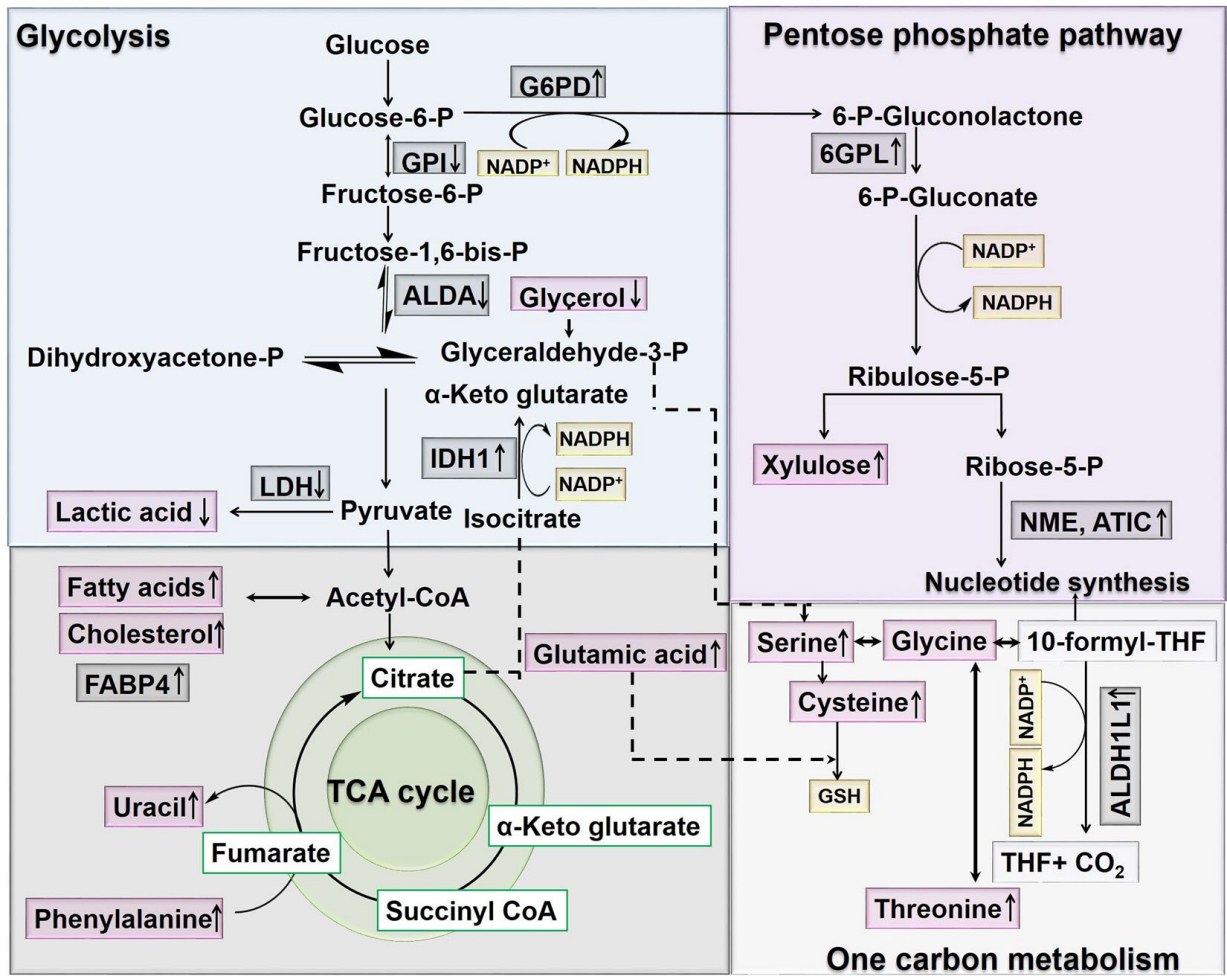
Schematic representation of the hypothesis proposed for the B[a]P-mediated metabolic flux redistribution. B[a]P exposure leads to the disturbance in the cellular redox state and hence to an increase in NADPH content of the cells by redirecting the metabolic flux towards the pentose phosphate pathway (PPP). It is indicated by the down-regulation of glycolytic proteins and an up-regulation of PPP proteins. The grey boxes represent the differentially regulated proteins identified by 2D BN/SDS-PAGE gel electrophoresis, whereas the pink boxes represent the metabolites identified by GC-MS analysis. Abbreviations: G6PD: glucose-6-phosphate dehydrogenase, GPI: glucose-6-phosphate isomerase isoform 1, ALD: fructose 1,6-bisphosphate, LDH: lactate dehydrogenase, FABP4: adipocyte fatty acid binding protein, 6GPL: 6-phosphogluconolactonase, NME: nucleoside diphosphate kinase, ATIC bifunctional purine biosynthesis protein, IDH1: cytosolic isocitrate dehydrogenase, ALDH1L1: 10-formyltetrahydrofolate dehydrogenase, THF: tetrahydrofolate, GSH: glutathione.

In our quest to determine the possible role of B[a]P in bladder toxicity, the combination of proteomic analyses of RT4 lysates by 2D BN/SDS-PAGE and subsequent MALDI-TOF-MS analysis in combination with a metabolomic approach provides detailed molecular insights into the orchestrated cellular response to B[a]P-induced oxidative stress after low-dose exposure. Following exposure of human bladder epithelial cells to 0.5 µM B[a]P the observed changes in protein alteration and metabolite levels point towards a metabolic flux redistribution that the cells undergo to avoid oxidative stress and to redirect the cellular metabolism toward detoxification of reactive B[a]P metabolites. Particularly, the repression of proteins involved in glycolysis and the up-regulation of proteins involved in the PPP are indicative of such a mechanism (Fig. 6, Table 1). Also, as indicated by the metabolomic data, it appeared that the cells were utilizing alternate substrates such as glutamic acid for tricarboxylic acid cycle function and amino acid generation (such as threonine, serine and cysteine), intermediates necessary for normal cell function regeneration and maintenance of redox balance. The study provides the snapshot of events which the cells undergo in the first 24 hrs after exposure to low concentration of B[a]P.

### Cytotoxicity of B[a]P in human bladder epithelial cells

In the risk assessment, we have to determine events that allow the differentiation of adaptive from adverse biological conditions. Thus, deriving insight into the cellular defence mechanism along a dose-response relation is a crucial prerequisite to identify potential biomarkers. To date, toxicological research regarded DNA damage and oxidative stress as key events in the causal chain leading to adverse effects in B[a]P-exposed cells. However, this paradigm bases on studies that used high dosage of B[a]P. In order to identify pathomechanism behind its toxicity, there is an urgent need to study the complex biological effects upon B[a]P-exposure to low doses.

In order to study the dose-response relation for B[a]P, RT4 cells were exposed 0.5 to 100 µM of B[a]P. One of the interesting observations made in this study was the absence/lack of the expected ROS production (Fig. 1A).

This was accompanied at lower B[a]P doses by a slight reduction in proliferation without any impact on cell death. At a concentration of 10 µM B[a]P and more, the proliferation was markedly slowed down and now the cells were prone to cell death (Fig. 1B,C). The slight decrease in proliferation as observed for the low concentration of B[a]P is an indication that cells allow for damage repair in order to return to normal cell cycling. However, on exposure to the high B[a]P concentration, this defense is overruled and cells undergo cell death. Therefore, we expected a comprehensive adaption of the cellular response to lower concentrations of B[a]P. To identify the molecular mechanism behind this, we combined metabolomic and proteomic analysis and exposed the cells to a representative concentration of the lower range, namely/more precisely 0.5 µM B[a]P.

### Proteomic and metabolomic analysis of B[a]P exposed human urinary bladder epithelial (RT4) cells

Till date, only a limited number of studies have applied proteomic approaches (preferentially 2D gel electrophoresis) to understand the toxicity of B[a]P^21^. In the present study we for the first time applied the 2D BN/SDS-PAGE technique in combination with metabolomics to elucidate the presence of protein-protein interactions that regulate cellular metabolism in B[a]P-exposed cells. As discussed in results section, MALDI-TOF-MS examination of differentially altered proteins indicated a down-regulation of proteins involved in the glycolysis (ALDA, GPI, LDHA) and an up-regulation of proteins involved in the PPP (G6PD, 6PGL) (Table 1). Other than this, an increased activity of proteins involved in oxidative phosphorylation (ATP synthase, H + transporting, mitochondrial F1 complex (ATP5A1), mitochondrial ATP synthase, H + transporting F1 complex beta subunit (ATP5B), NADH dehydrogenase iron-sulfur protein (NDUFV1), the ones involved in generation of reducing agent nicotinamide adenine dinucleotide phosphate (NADPH)-producing dehydrogenases (isocitrate dehydrogenase (cytosolic, IDH1), 10-formyltetrahydrofolate dehydrogenase (ALDH1L1) (Suppl. Table 1) and an antioxidant enzyme (peroxiredoxin-6, PRDX6) was also observed. When the biological networks of the identified and differentially expressed proteins were analyzed by using the STITCH software, the proteins were grouped PPP, one carbon metabolism and nucleotide biosynthetic pathways (Fig. 3A,B).

Convincingly, the proteomic analysis of cells exposed to B[a]P highlighted various cellular pathways which were altered to maintain redox homeostasis of the cells. To gather a more sophisticated overview of ongoing processes we extended our research towards metabolic profiling to address cellular pathways, which further support our proteomic approach. By application of gas chromatography mass spectrometry we were able to further observe multiple alterations in metabolic pathway linked to synthesis of essential building blocks (i.e. amino acid, lipids, and nucleotides) (Fig. 5). It appeared like indicated from proteomic analysis, part of the energetic substrates were redirected into metabolic routes to generate antioxidant molecules such as NADPH, GSH and metabolites for DNA replication/repair (Suppl. Tables 2 and 3), the details of which will be discussed in the following sections.

### Up-regulation of pathways involved in maintaining ROS homeostasis

Other than its mutagenic and carcinogenic properties, B[a]P has been well documented to cause ROS-realted toxicity^22, 23^. An increased ROS level is known to cause wide-range of damages, including cell death and to protect themselves against these damages, the cells alters the expression of genes encoding antioxidants and other metabolic enzymes^24^.

The pentose phosphate pathway (also known as hexose monophosphate shunt) is an alternative to the glycolytic pathway, where glucose when present in large amounts is metabolized to intermediates taking part in nucleotide synthesis and NADPH generation^25^. NADPH plays a critical role in maintaining the cellular antioxidant capacity by regenerating ROS scavengers, the reduced pools of GSH and thioredoxin (TRX)^26^. Dynamic rerouting of the metabolic flux to the pentose phosphate pathway, as a conserved post-translational response to oxidative stress has been recently reported in several studies^27, 28^. Interestingly, for the proteins differentially regulated in B[a]P-exposed RT4 cells, two of the up-regulated proteins (G6PD, 6PGL) were related to the pentose phosphate pathway system (Fig. 6).

Overexpression of glucose-6-phosphate dehydrogenase (G6PD), the rate limiting enzyme of the PPP is known to enhance PPP- dependent production of NADPH and GSH^29–31^. An increased enzyme activity of this enzyme by 85% in B[a]P-exposed cells as compared to the control cells indicated toward such flux (Fig. 4B). Further, as discussed in the result section, the increased flux into PPP generates 6-phosphogluconic acid (6-PGA) which is an inhibitor of GPI, one of the enzyme, which was found to be downregulated in our proteomic results. However, analysis of GPI activity in B[a]P exposed cells revealed similar levels as in control cells (Fig. 4B).

On the other hand, the analysis of cytosolic NADPH/NADP^+^ ratio did not reveal much difference between control and B[a]P exposed cells. From the results, we can only speculate that it is because of its continuous consumption in ROS clearance mechanisms and in maintaining GSH/GSSG redox balance. Moreover, because of NADPH continuous utilization, its inhibitory effect on G6PD is reduced, thus, increasing its activity and thereby promoting the glucose flux from glycolysis into PPP. On the other hand, the whole cell NADPH/NADP^+^ ratio difference is a product of changes in the cytosol as well as other compartments of the cells i.e. mitochondria, endoplasmic reticulum, which was clearly visible during proteomic analysis of B[a]P-exposed cells (Fig. 3). Moreover, the shift to PPP in exposed cells also leads to an accumulation of xylulose, as observed in our metabolomic data (Table 3) which in addition with ribulose-5-phosphate 3-epimerase aids to protect the cells against oxidative stress^32^.

An up-regulation of proteins and metabolites involved in another important pathway of the antioxidant defense system as pointed by STITCH analysis was the serine-glycine one carbon metabolism (Fig. 3B). This pathway is known for its regulation of nucleic acids, lipids and protein synthesis and most importantly antioxidant defense systems via generation of NADPH^33^. Two steps in the one-carbon cycle have the potential to generate NADPH. The first is the oxidation of 5,10-methylene-tetrahydrofolate by cytosolic and mitochondrial methylenetetrahydrofolate dehydrogenase. The second NADPH-producing step in the folate cycle depends on cytosolic and mitochondrial 10-formyl-tetrahydrofolate dehydrogenases (ALDH1L1 and ALDH1L2 respectively), which catalyze reactions that ‘waste’ the one-carbon unit in 10-formyl-THF by releasing it as CO_2_ while generating NADPH^34^. An up-regulation of ALDH1L1 upon B[a]P exposure was observed in our studies. Further, the analysis of water-soluble metabolites during our metabolomic analysis also revealed increased levels of amino acids such as serine, cysteine, isoleucine, proline, alanine and glutamic acid (Tables 2 and 3). Several recent findings suggest that serine is preferentially utilized in mitochondria of mammalian cells to generate NADPH^35, 36^. Other than serine, glutamic acid which is used by cells to generate glutamate which is subsequently used to fuel TCA cycle via glutaminolysis is also recognized to increases glutathione synthesis via promoting the uptake of cysteine^37^, one of the main contributors of GSH synthesis.

Other than these two pathways, alteration in several mitochondrial proteins were also observed. Mitochondria are known as the key organelle responsible for the regulation of redox signaling and redox homeostasis of cells and are known to directly or indirectly control a number of cellular processes including proliferation, ATP generation and cell death^38^. An up-regulation of proteins involved in oxidative phosphorylation (ATP5A1, ATP5B, 3NDUFV1) as observed by proteomic analysis indicates towards the shift the oxidative phosphorylation for ATP synthesis under limited glucose conditions (Fig. 3A).

Furthermore, it appeared that the cells were utilizing alternate substrates such as glycerol, fatty acid (stearic, linolenic, oleic), and glutamic acid (as observed in metabolomic data, Tables 2 and 3) for tricarboxylic acid cycle function to sustain mitochondrial ATP generation and further generation of NADPH and GSH (Fig. 6). The breakdown of fatty acid generates acetyl-CoA and reducing equivalents NADH and FADH_2_ which are used by the electron transport chain (ETC) to produce ATP and also for generation of citrate which is exported into cytosol and oxidatively decarboxylated by IDH1 and malic enzyme producing NADPH^39^. Nevertheless, the cellular membrane with its high content of unsaturated fatty acids plays a protective, anti-inflammatory role and indirectly an antioxidant role, favoring physiological defense processes against free radicals^40^.

### Cellular response to macromolecule damage

In our previous study with primary porcine urinary bladder epithelial cells, B[a]P was able to cause not only single-strand breaks as reported by many other studies but also more hazardous double-strand breaks^9^. In the present study, the proteomic analysis of the nuclear fraction revealed down-regulations of different histone proteins (Suppl. Table 3). Such results were in accordance with other studies describing alterations of histone proteins following DNA damage due to inhibition of cyclin-dependent kinase 2 activity^41^. The cellular response to DNA damage and repair was further seen by alterations of proteins involved in the generation of intermediates for DNA repair (nucleoside diphosphate kinase, NME2, and bifunctional purine biosynthesis protein, ATIC)^42^ and protein biosynthesis for repair of damage due to chromosome aberrations (T-complex protein 1 subunit beta isoform 2 (TCP1), CCT2, VCP, PDIA4, cyclophilin B (PPIB), cyclophilin A (PPIA), calreticulin (CALR) (Suppl. Tables 1 and 2). An up-regulation of metabolites involved in biosynthetic pathways such as protein biosynthesis (isoleucine, proline, alanine and glutamic acid) and DNA synthesis (uracil) was also observed during our metabolomic analysis (Tables 2 and 3).

### Hypothesis: Redirection of the metabolic flux of cells from glycolysis to the pentose phosphate pathway

We assume that in order to avoid B[a]P-induced toxicity, the low level of ROS molecules activates signaling networks such as transcription factor nuclear factor erythroid 2-related factor 2 (Nrf2) hypoxia-inducible factor (HIF)1α and nuclear factor kappa-light-chain-enhancer of activated B cells (NFκB), which are involved in maintaining cell homeostasis^43, 44^. Once activated, Nrf2 binds to antioxidant response elements (ARE) of antioxidant genes including genes for NADPH production, glutathione homeostasis/utilization and of phase I and II enzymes^45, 46^. As a result of which the cells redirected the cellular metabolism from glycolysis to PPP (as observed in our proteomic and metabolomic results) to increase the production of reducing equivalents essential for the activation of antioxidant enzymes and the reduction of glutathione, both necessary for the detoxification of reactive B[a]P metabolites (Fig. 6).

The rerouting of the glycolytic pathway impairs cell proliferation (as observed in our study too,) and enables the cell to repair stress-mediated macromolecule damage and to conserve reducing equivalents for cell protection. This change in cellular behavior is referred as reverse Warburg effect for cancer cells. During oxidative stress it is supposed that adjacent stromal fibroblasts activate the two transcription factors (HIF)1α and NFκB which produce recycled nutrients (e.g. lactic acid and glutamine), ready to be transferred and used to support the tricarboxylic acid cycle (TCA) in adjacent cells and to protect them from cell death^47^. This assumption was supported by the increased NADPH (cytosolic) (Fig. 4E,F) and GSH content (Suppl. Fig. 1) of the cells exposed to B[a]P, along with the alteration of cytosolic proteins such as IDH1 and ALDH1L1, which play a significant role in NADPH production (Suppl. Table 1 and Fig. 4) and up-regulation of protein and metabolites involved in protein and nucleotide biosynthesis. Together the results point towards a rerouting of the carbohydrate flux that the bladder epithelia cells undergo upon exposure to B[a]P to avoid the deleterious consequences of oxidative stress.

## Conclusions

The study provides new insights into a B[a]P-induced shift in cellular metabolism towards processes involved in NADPH generation. B[a]P exposure causes oxidative DNA damage and hence cellular perturbations. To overcome these effects, the cells undergo a metabolic flux change from glycolysis to the pentose phosphate pathway. This shift leads to the generation of the redox cofactor NADPH that is essential for the activity of many antioxidant enzymes and intermediates necessary for the de novo generation of nucleotides (purine and pyrimidine) and for the normal functioning of the cells. The study provides preliminary indication of changes in cellular metabolism upon B[a]P exposure, however, further investigations are required to confirm our hypothesis.

## Electronic supplementary material

Supplemetary information
