# Supplementary material for: Benzo[a]pyrene-induced metabolic shift from glycolysis to pentose phosphate pathway in the human bladder cancer cell line RT4
Source: Sci Rep. 2017 Aug 29;7:9773. doi: 10.1038/s41598-017-09936-1 (PMC5575001; doi:10.1038/s41598-017-09936-1)

**Supplementary Data**

**Benzo[a]pyrene-induced metabolic shift from glycolysis to pentose phosphate pathway in human bladder cancer cell line (RT4)**

***Nisha Verma*, Mario Pink, Stefan Boland, Albert W. Rettenmeier, and Simone Schmitz-Spanke***

*****Correspondence:**

Nisha Verma, Institute and Outpatient Clinic of Occupational, Social and Environmental Medicine, University of Erlangen-Nuremberg, Schillerstraße 25/29, D-91054 Erlangen, Germany

**E-mail:** [nishaverma24@gmail.com](mailto:nishaverma24@gmail.com)

**Fax:** +49-9131-8522317

**Supplementary Figure 1** Analysis of the intracellular glutathione content. After 24 h of exposure with B[a]P (0.5 µM), the GSH content was quantified in the supernatant obtained from RT4 cell homogenates. The measurements were performed by using an ELISA plate reader (OD_412nm_). The data were presented as mean ± standard of mean of four independent experiments with different RT4 cell lysates. The level of significance relative to the control was determined by using the t-test (*p <0.05).

**
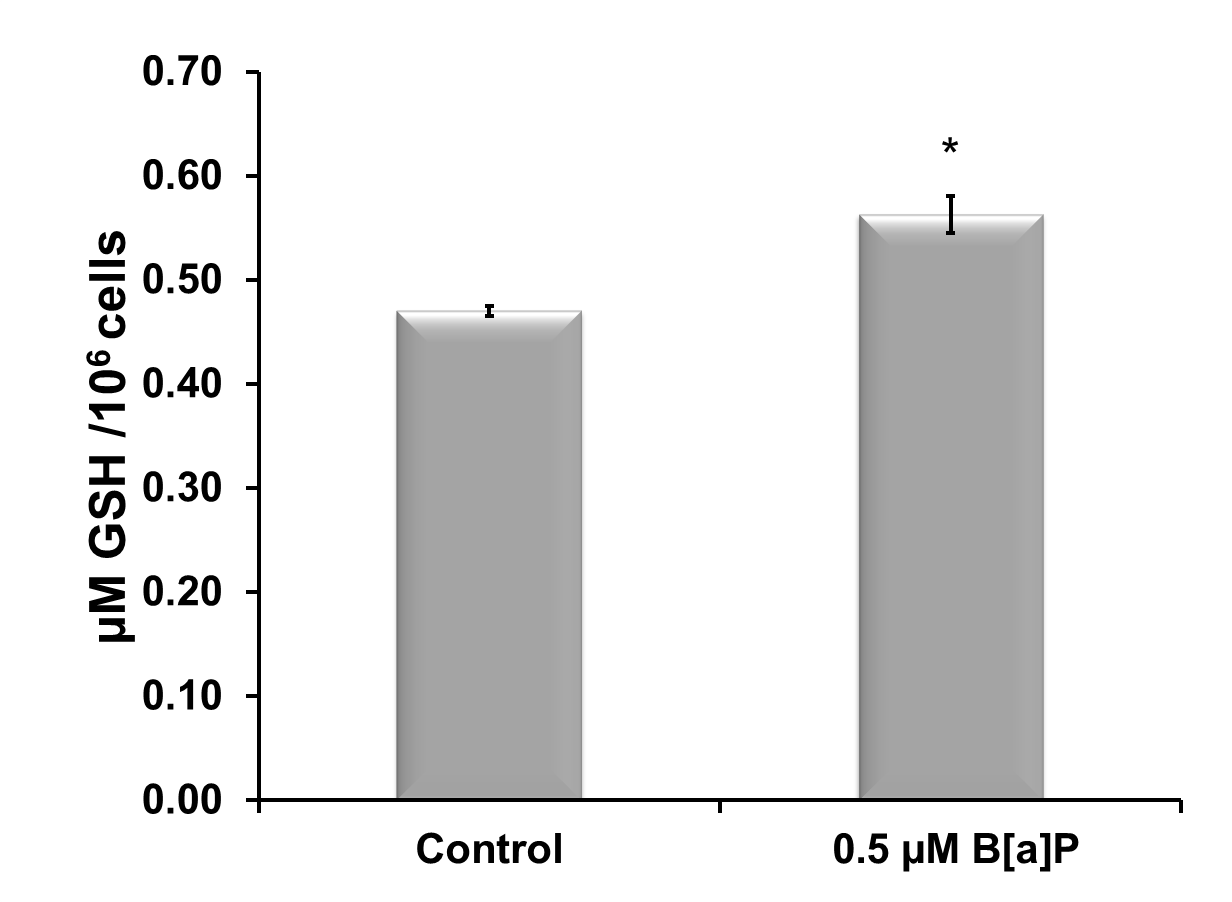
**

**Supplementary Table 1**: List of proteins differentially expressed in the cytosolic fraction of RT4 cells identified by MALDI-TOF-MS after exposure to 0.5 µM B[a]P for 24 h (see gel with ID numbers in Figure 1A).

| **^1^Spot no** | **^2^Gene name** | **^2^Protein name** | **^3^Score** | **Sequence coverage** | **Peptide matches** | **Control**^*^ | **B[a]P exposed**^*^ | **^4^Regulation** | **^5^T-test** |
| --- | --- | --- | --- | --- | --- | --- | --- | --- | --- |
|  |  |  |  |  |  |  |  |  |  |
| 4 | PFN1 | human platelet profilin complexed with L-Pro10-iodotyrosine peptide | 147 | 58 | 12/21 | 0.27026 | 0.1516 | -1.78267 | 99.83655 |
| 16 | G6PD | glucose-6-phosphate dehydrogenase | 161 | 24 | 23/50 | 0.1912 | 0.36028 | 1.88427 | 98.57494 |
| 18 | VCP | transitional endoplasmic reticulum ATPase | 234 | 46 | 32/50 | 0.1136 | 0.21871 | 1.92522 | 97.22852 |
| 17 | ALDH1L1 | 10-formyltetrahydrofolate dehydrogenase | 423 | 51 | 40/50 | 0.1023 | 0.22473 | 2.19610 | 99.25462 |
| 14 | 6PGL | 6-phosphogluconolactonase | 168 | 17 | 23/40 | 0.8902 | 0.38719 | 2.29904 | 97.70821 |
| 21 | CCT2 | T-complex protein 1 subunit beta isoform 2 | 131 | 44 | 18/50 | 0.1214 | 0.25756 | 2.12253 | 99.03545 |
| 11 | IDH1 | human cytosolic NADP(+)-dependent isocitrate dehydrogenase in complex with NADP^+^ and isocitrate | 240 | 53 | 21/50 | 0.04149 | 0.08076 | 1.94645 | 99.4232 |
| 5 | NME2 | nucleoside diphosphate kinase | 154 | 73 | 11/24 | 0.02297 | 0.06192 | 2.69598 | 96.05284 |
| 3 | FABP4 | human adipocyte fatty acid binding protein | 141 | 69 | 12/24 | 0.0999 | 0.41279 | 4.13207 | 97.86784 |
| 14 | ALDA | human fructose 1,6-bisphosphate aldolase complexed with fructose 1,6-bisphosphate | 293 | 66 | 22/28 | 1.61613 | 0.88703 | -1.82195 | 99.48823 |
| 15 | GPI | glucose-6-phosphate isomerase isoform 1 | 123 | 27 | 14/25 | 0.78628 | 0.25837 | -3.04320 | 99.22896 |
| 12 | LDHA | lactate dehydrogenase A variant | 113 | 31 | 16/26 | 0.08744 | 0.0715 | -1.22291 | 99.50999 |
| 6 | PRDX6 | peroxiredoxin-6 | 92 | 48 | 10/50 | 0.0639 | 0.13143 | 2.05677 | 95.58708 |
| 19 | TCP1 | T-complex protein 1 subunit alpha isoform a | 148 | 31 | 18/50 | 0.1295 | 0.46505 | 3.59029 | 95.13259 |
| 8 | PRDX6 | peroxiredoxin-6 | 153 | 64 | 14/50 | 0.1626 | 0.07141 | -2.27703 | 98.31438 |
| 25 | ATIC | bifunctional purine biosynthesis protein PURH | 203 | 50 | 22/50 | 0.04921 | 0.14598 | 2.96631 | 97.16220 |

**^1^**: Experimental ID number.

**^2^**: Gene name and protein name entries in Uniprot database.

**^3^**: Score and peptides matches in Mascot search engine, protein scores greater than 65 are considered significant (p <0.05).

**^4^**: The values represent the ratio of the relative spot volume of treated and control cells as determined by using the Delta2D v4.0 software.

**^5^**: t-tests value (*q*) shown by Delta2D v4.0 software is derived from the *p*-value (*q* = (1 – *p*)×100).

^*^: Values represent spot volume as determined by using Delta2D v4.0 software.

**Supplementary Table 2**: List of proteins differentially expressed in the membrane/organelle fraction of RT4 cells identified by MALDI-TOF-MS after exposure to 0.5 µM B[a]P for 24 h (see gel with ID numbers in Figure 1B).

| **^1^Spot no** | **^2^Gene name** | **^2^Protein name** | **^3^Score** | **Sequence coverage** | **Peptide matches** | **Control**^*^ | **B[a]P exposed**^*^ | **^4^Regulation** | **^5^T-test** |
| --- | --- | --- | --- | --- | --- | --- | --- | --- | --- |
|  |  |  |  |  |  |  |  |  |  |
| 06 | CLTC | clathrin heavy chain 1 isoform 6 | 342 | 27 | 39/50 | 0.25681 | 0.44499 | 1.73274 | 98.86489 |
| 15 | PDIA4 | protein disulfide-isomerase A4 precursor | 329 | 47 | 33/50 | 0.14801 | 0.35479 | 2.39697 | 99.46539 |
| 23 | UGDH | UDP-glucose 6-dehydrogenase isoform 1 | 167 | 48 | 19/42 | 0.50683 | 0.92322 | 1.82155 | 95.0701 |
| 30 | ANXA10 | annexin A10 | 98 | 30 | 14/50 | 0.20344 | 0.05144 | -3.95467 | 92.3265 |
| 33 | TPM3 | tropomyosin alpha-3 chain isoform 3 | 103 | 45 | 22/50 | 0.26642 | 0.49472 | 1.85692 | 95.92751 |
| 39 | PPIB | cyclophilin B complexed with cyclosporin | 116 | 57 | 14/37 | 0.44698 | 0.78213 | 1.74984 | 99.99252 |
| 43 | PPIA | cyclophilin A complexed with dipeptide gly-pro | 156 | 67 | 11/14 | 0.0682 | 0.23274 | 3.41278 | 99.02547 |
| 07 | UBA1 | ubiquitin-like modifier-activating enzyme 1 | 349 | 46 | 36/50 | 0.12823 | 0.34286 | 2.67377 | 96.9292 |
| 16 | P4HA1 | procollagen-proline, 2-oxoglutarate 4-dioxygenase | 251 | 57 | 20/31 | 0.02596 | 0.20304 | 7.82073 | 98.80403 |
| 18 | UBA1 | ubiquitin-like modifier-activating enzyme 1 | 287 | 34 | 32/50 | 0.1947 | 0.1111 | -1.7525 | 99.70596 |
| 19 | CALR | calreticulin precursor variant | 125 | 43 | 11/17 | 0.12448 | 0.26813 | 2.15407 | 98.48998 |
| 22 | ATP5A1 | ATP synthase, H+ transporting, mitochondrial F1 complex | 182 | 33 | 17/32 | 0.14728 | 0.3747 | 2.54415 | 98.70618 |
| 24 | ATP5B | mitochondrial ATP synthase, H+ transporting F1 complex beta subunit | 155 | 47 | 20/50 | 0.16234 | 0.29685 | 1.82857 | 99.04031 |
| 31 | GNPDA1 | glucosamine-6-phosphate isomerase 1 | 150 | 64 | 14/23 | 0.03817 | 0.08584 | 2.24889 | 97.8611 |
| 08 | UBA1 | ubiquitin-like modifier-activating enzyme 1 | 331 | 37 | 30/36 | 0.10308 | 0.26797 | 2.59967 | 96.11044 |
| 42 | PPIA | cyclophilin A complexed with dipeptide gly-pro | 134 | 82 | 11/14 | 0.065 | 0.24331 | 3.74312 | 96.28853 |
| 45 | PPIA | cyclophilin A complexed with dipeptide gly-pro | 157 | 75 | 15/32 | 0.12654 | 0.38965 | 3.07921 | 90.85715 |
| 48 | KRT9 | keratin, type I cytoskeletal 9 | 108 | 41 | 17/50 | 0.07488 | 0.1966 | 2.62558 | 98.96328 |

**^1^**: Experimental ID number.

**^2^**: Gene name and protein name entries in Uniprot database.

**^3^**: Score and peptides matches in Mascot search engine, protein scores greater than 65 are considered significant (p <0.05).

**^4^**: The values represent the ratio of the relative spot volume of treated and control cells as determined by using the Delta2D v4.0 software.

**^5^**: t-tests value (*q*) shown by Delta2D v4.0 software is derived from the *p*-value (*q* = (1 – *p*)×100).

^*^: Values represent spot volume as determined by using Delta2D v4.0 software.

S**upplementary Table 3**: List of proteins differentially expressed in the nuclear fraction of RT4 cells identified by MALDI-TOF-MS after exposure to 0.5 µM B[a]P for 24 h (see gel with ID numbers in Figure 1C).

| **^1^Spot no** | **^2^Gene name** | **^2^Protein name** | **^3^Score** | **Sequence coverage** | **Peptide matches** | **Control**^*^ | **B[a]P exposed**^*^ | **^4^Regulation** | **^5^T-test** |
| --- | --- | --- | --- | --- | --- | --- | --- | --- | --- |
|  |  |  |  |  |  |  |  |  |  |
| 2 | KRT8 | cytokeratin 8 | 261 | 77 | 23/45 | 2.41674 | 1.26769 | -1.90642 | 99.19725 |
| 3 | KRT7 | keratin, type II cytoskeletal 7 | 330 | 53 | 18/22 | 1.28027 | 0.65476 | -1.95532 | 99.77895 |
| 4 | KRT19 | keratin, type I cytoskeletal 19 | 416 | 74 | 35/47 | 0.45543 | 0.16297 | -2.79450 | 98.8258 |
| 5 | HNRPA1 | heterogeneous nuclear ribonucleoprotein A1 | 96 | 42 | 11/50 | 0.68984 | 1.83215 | 2.65593 | 99.89055 |
| 6 | VDAC1 | porin 31HM | 107 | 53 | 11/50 | 0.89633 | 2.0858 | 2.32705 | 99.80842 |
| 7 | HIST1H1B | histone H1.5 | 100 | 43 | 10/32 | 0.78077 | 3.16297 | 4.05107 | 99.78925 |
| 8 | PHB | prohibitin | 77 | 38 | 27/50 | 0.43339 | 1.75033 | 4.03869 | 99.93215 |
| 9 | NDUFV1 | NADH dehydrogenase iron-sulfur protein 3 | 70 | 27 | 14/50 | 0.06974 | 0.60944 | 8.73903 | 98.34015 |
| 10 | HECTD3 | E3 ubiquitin-protein ligase | 75 | 77 | 29/50 | 0.14277 | 1.24668 | 8.73204 | 98.56757 |
| 11 | RPL10A | 60S ribosomal protein L10a | 77 | 50 | 10/50 | 0.22796 | 0.48694 | 2.13607 | 97.43598 |
| 15 | UBE2K | E2-25k and Ubb+1 Complex | 79 | 59 | 20/45 | 0.02914 | 0.10524 | 3.61185 | 95.22588 |
| 22 | PSMB9 | proteasome subunit, beta type, 9 | 74 | 32 | 10/44 | 3.35729 | 7.645 | 2.27713 | 99.4469 |
| 23 | HIST1H2BM | histone H2B type 1-M | 76 | 50 | 34/50 | 10.0317 | 2.61843 | -3.83119 | 97.73065 |
| 24 | HIST1H2AG | histone H2A type 1-B/E | 70 | 30 | 24/40 | 3.87231 | 0.93108 | -4.15894 | 99.32237 |
| 25 | HIST2H3A | H3-H4 chaperone asf1 bound to histones H3 And H4 | 120 | 66 | 32/50 | 3.59978 | 1.1384 | -3.16213 | 99.99695 |
| 26 | MYH9 | myosin-9 | 253 | 23 | 40/50 | 0.04939 | 0.1515 | 3.06755 | 99.80663 |
| 27 | DHX9 | nuclear DNA helicase II | 152 | 19 | 25/50 | 0.34593 | 1.07541 | 3.10872 | 98.59242 |

**^1^**: Experimental ID number.

**^2^**: Gene name and protein name entries in Uniprot database.

**^3^**: Score and peptides matches in Mascot search engine, protein scores greater than 65 are considered significant (p <0.05).

**^4^**: The values represent the ratio of the relative spot volume of treated and control cells as determined by using the Delta2D v4.0 software.

**^5^**: t-tests value (*q*) shown by Delta2D v4.0 software is derived from the *p*-value (*q* = (1 – *p*)×100).

^*^: Values represent spot volume as determined by using Delta2D v4.0 software

**Supplementary Table 4: List of positively identified metabolites**

|  | **Retension time** | **Accession** | **Name** | **Formula** | **Mass** | **Score** | **Hit** |
| --- | --- | --- | --- | --- | --- | --- | --- |
|  | 6.1139 | PR010200 | Linolenic acid | C18H30O2 | 278.22458 | 0.508 | 37 |
|  | 6.6951 | PR010225 | Cysteamine | C2H7NS | 77.02992 | 0.7501 | 13 |
|  | 6.8795 | KZ000258 | Oxalic acid | C2H2O4 | 89.99531 | 0.7876 | 8 |
|  | 7.4879 | OUF00458 | (-)-Shikimic acid | C7H10O5 | 174.05282 | 0.4814 | 18 |
|  | 7.7642 | OUF00338 | Malonic acid | C3H4O4 | 104.01096 | 0.7024 | 4 |
|  | 8.3469 | PR010059 | L-Glutamine | C5H10N2O3 | 146.06914 | 0.45 | 26 |
|  | 8.3928 | OUF00362 | N-Acetyl-DL-alanine | C5H9NO3 | 131.05824 | 0.6351 | 24 |
|  | 10.7817 | OUF00033 | 2-Hydroxypyridine | C5H5NO | 95.03711 | 0.9714 | 15 |
|  | 12.8341 | OUF00154 | D-3-Phenyllactic acid | C9H10O3 | 166.06299 | 0.5498 | 16 |
|  | 12.8521 | OUF00314 | L-(+)-Lactic acid | C3H6O3 | 90.03169 | 0.9345 | 30 |
|  | 12.8845 | OUF00501 | beta-N-Methyl-amino-L-alanine | C4H10N2O2 | 118.07423 | 0.4842 | 16 |
|  | 13.2429 | OUF00125 | Benzen-1,3-Dicarboxylic acid | C8H6O4 | 166.02661 | 0.5908 | 14 |
|  | 14.1659 | PR010063 | L-Methioninesulfone | C5H11NO4S | 181.04088 | 0.3806 | 9 |
|  | 15.1311 | OUF00053 | 3-Hydroxyisovaleric acid | C5H10O3 | 118.06299 | 0.6865 | 10 |
|  | 15.6815 | OUF00028 | 2'-Deoxyinosine | C10H12N4O4 | 252.08585 | 0.4024 | 30 |
|  | 17.5509 | OUF00378 | N-Acetyl-Serotonin | C12H14N2O2 | 218.10553 | 0.3931 | 18 |
|  | 17.5509 | PR010172 | L-Asparagine | C4H8N2O3 | 132.05349 | 0.381 | 22 |
|  | 18.2352 | OUF00184 | DL-3,4-Dihydroxymandelic acid | C8H8O5 | 184.03717 | 0.3785 | 14 |
|  | 18.2563 | PR010030 | L-(+)-Arginine | C6H14N4O2 | 174.11168 | 0.3247 | 7 |
|  | 18.5868 | OUF00428 | Propyleneglycol | C3H8O2 | 76.05243 | 0.4285 | 10 |
|  | 18.8014 | OUF00031 | 2'-Deoxyuridine | C9H12N2O5 | 228.07462 | 0.4474 | 18 |
|  | 19.5076 | PR010237 | Stearic acid | C18H36O2 | 284.27153 | 0.4708 | 11 |
|  | 19.9443 | PR010176 | Malonic acid | C3H4O4 | 104.01096 | 0.5354 | 7 |
|  | 20.4909 | PR010225 | Cysteamine | C2H7NS | 77.02992 | 0.8537 | 27 |
|  | 20.6414 | PR010153 | DL-beta-Hydroxybutyric acid | C4H8O3 | 104.04734 | 0.5515 | 10 |
|  | 20.7566 | PR010109 | 3-Indoleacetonitrile | C10H8N2 | 156.06875 | 0.4965 | 11 |
|  | 21.2964 | OUF00127 | Benzoic acid | C7H6O2 | 122.03678 | 0.9421 | 9 |
|  | 21.7828 | OUF00237 | Gallic acid | C7H6O5 | 170.02152 | 0.5658 | 22 |
|  | 22.0132 | PR010030 | L-(+)-Arginine | C6H14N4O2 | 174.11168 | 0.4936 | 21 |
|  | 22.1291 | OUF00383 | Octanoic acid | C8H16O2 | 144.11503 | 0.8375 | 18 |
|  | 22.3309 | KZ000059 | Mesaconic acid | C5H6O4 | 130.02661 | 0.4792 | 6 |
|  | 22.3949 | KZ000132 | Ethanolamine | C2H7NO | 61.05276 | 0.9095 | 14 |
|  | 22.7766 | OUF00438 | Pyrophosphoric acid | H4O7P2 | 177.94323 | 0.9187 | 36 |
|  | 22.9603 | KZ000140 | Glycerol | C3H8O3 | 92.04734 | 0.9472 | 38 |
|  | 23.2983 | KZ000069 | Psoralen | C11H6O3 | 186.03169 | 0.3905 | 7 |
|  | 23.5317 | OUF00385 | N-FormylGlycine | C3H5NO3 | 103.02694 | 0.6413 | 7 |
|  | 23.6522 | PR010135 | Ribitol | C5H12O5 | 152.06847 | 0.696 | 33 |
|  | 23.7124 | PR010238 | Succinic acid | C4H6O4 | 118.02661 | 0.8349 | 34 |
|  | 24.1642 | OUF00398 | N-alpha-Acetyl-L-ornithine | C7H14N2O3 | 174.10044 | 0.4021 | 48 |
|  | 24.8335 | OUF00392 | Pelargonic acid | C9H18O2 | 158.13068 | 0.8698 | 25 |
|  | 25.2212 | KZ000161 | L-Serine | C3H7NO3 | 105.04259 | 0.5709 | 9 |
|  | 25.2543 | KZ000124 | D-(+)-Trehalose | C12H22O11 | 342.11621 | 0.4134 | 8 |
|  | 25.2543 | OUF00078 | 5-HydroxymethylUracil | C5H6N2O3 | 142.03784 | 0.399 | 14 |
|  | 25.2724 | KZ000124 | D-(+)-Trehalose | C12H22O11 | 342.11621 | 0.5175 | 18 |
|  | 25.5412 | OUF00282 | Inosine-3',5'-cyllic-monophosphate | C10H11N4O7P | 330.03654 | 0.2829 | 18 |
|  | 25.9508 | PR010024 | Pyridoxamine | C8H12N2O2 | 168.08988 | 0.3281 | 12 |
|  | 26.3490 | OUF00239 | Gentisic acid | C7H6O4 | 154.02661 | 0.5819 | 25 |
|  | 26.7247 | PR010109 | 3-Indoleacetonitrile | C10H8N2 | 156.06875 | 0.5772 | 38 |
|  | 27.9368 | PR010100 | Uracil | C4H4N2O2 | 112.02728 | 0.3583 | 27 |
|  | 28.2500 | OUF00088 | 7,8-Dihydroneopterin | C9H13N5O4 | 255.09675 | 0.2408 | 5 |
|  | 28.2817 | OUF00374 | N-Acetylneuraminic acid | C11H19NO9 | 309.10598 | 0.4239 | 17 |
|  | 28.8907 | OUF00031 | 2'-Deoxyuridine | C9H12N2O5 | 228.07462 | 0.3296 | 13 |
|  | 29.0436 | KZ000012 | Adenine | C5H5N5 | 135.0545 | 0.5487 | 18 |
|  | 29.5397 | OUF00162 | DethioBiotin | C10H18N2O3 | 214.13174 | 0.7515 | 11 |
|  | 29.9094 | PR010228 | Psoralen | C11H6O3 | 186.03169 | 0.4452 | 26 |
|  | 30.1488 | OUF00282 | Inosine-3',5'-cyllic-monophosphate | C10H11N4O7P | 330.03654 | 0.4399 | 25 |
|  | 30.7240 | OUF00475 | trans-Chalcone | C11H15N5O4 | 281.1124 | 0.4707 | 7 |
|  | 31.1539 | OUF00300 | Lauric acid | C12H24O2 | 200.17763 | 0.8438 | 23 |
|  | 31.7690 | OUF00021 | 2-Aminopimelic acid | C7H13NO4 | 175.08446 | 0.5189 | 12 |
|  | 32.1801 | KZ000056 | Linoleic acid | C18H32O2 | 280.24023 | 0.5003 | 35 |
|  | 32.2004 | OUF00010 | 1-Methyluracil | C6H6N2O4 | 170.03276 | 0.3354 | 16 |
|  | 32.2004 | OUF00010 | 1-Methyluracil | C6H6N2O4 | 170.03276 | 0.3477 | 19 |
|  | 32.4142 | KZ000253 | Nonadecanoic acidmethylester | C20H40O2 | 312.30283 | 0.6883 | 58 |
|  | 32.7568 | OUF00475 | trans-Chalcone | C11H15N5O4 | 281.1124 | 0.5543 | 5 |
|  | 32.9510 | OUF00434 | Pyridoxal | C8H9NO3 | 167.05824 | 0.4978 | 24 |
|  | 32.9819 | PR010194 | DL-6,8-Thioctic acid | C8H14O2S2 | 206.04352 | 0.3225 | 21 |
|  | 32.9819 | OUF00291 | Kojic acid | C6H6O4 | 142.02661 | 0.6263 | 14 |
|  | 33.0391 | OUF00255 | Glycerol-2-phosphate | C3H9O6P | 172.01367 | 0.8042 | 34 |
|  | 33.3538 | PR010099 | Anthranilic acid | C7H7NO2 | 137.04768 | 0.6286 | 10 |
|  | 33.5706 | PR010126 | 2'-Deoxyadenosine | C10H13N5O3 | 251.10184 | 0.7988 | 4 |
|  | 33.6678 | KZ000210 | rac-Glycerol3-phosphate | C3H9O6P | 172.01367 | 0.8343 | 47 |
|  | 33.8846 | OUF00402 | O-Phospho ethanol amine | C2H8NO4P | 141.01909 | 0.7858 | 34 |
|  | 34.2000 | KZ000253 | Nonadecanoic acidmethylester | C20H40O2 | 312.30283 | 0.5946 | 25 |
|  | 34.4688 | OUF00262 | Heptadecanoic acid | C17H34O2 | 270.25588 | 0.4251 | 16 |
|  | 35.4566 | PR010196 | Fusaric acid | C10H13NO2 | 179.09463 | 0.5815 | 67 |
|  | 35.8955 | KZ000253 | Nonadecanoic acidmethylester | C20H40O2 | 312.30283 | 0.6852 | 67 |
|  | 36.3247 | OUF00006 | 1-Hexadecanol | C16H34O | 242.26097 | 0.7285 | 11 |
|  | 36.4504 | OUF00410 | Paeonol | C9H10O3 | 166.06299 | 0.3743 | 5 |
|  | 36.6093 | OUF00031 | 2'-Deoxyuridine | C9H12N2O5 | 228.07462 | 0.308 | 18 |
|  | 36.7011 | PR010239 | Thymine | C5H6N2O2 | 126.04293 | 0.4874 | 20 |
|  | 37.5511 | OUF00412 | Palmitoleic acid | C16H30O2 | 254.22458 | 0.8509 | 113 |
|  | 37.6339 | PR010057 | L-(-)-Cystine | C6H12N2O4S2 | 240.02385 | 0.2868 | 17 |
|  | 37.8289 | PR010133 | 3',5'-CyclicAMP | C10H12N5O6P | 329.05252 | 0.3936 | 36 |
|  | 37.8884 | PR010202 | Palmitic acid | C16H32O2 | 256.24023 | 0.8335 | 64 |
|  | 38.2694 | KZ000253 | Non adecanoic acidmethyl ester | C20H40O2 | 312.30283 | 0.3824 | 14 |
|  | 38.6563 | OUF00096 | Adenine | C5H5N5 | 135.0545 | 0.6395 | 83 |
|  | 38.7015 | KZ000035 | Hypoxanthine | C5H4N4O | 136.03851 | 0.5014 | 61 |
|  | 38.7369 | OUF00096 | Adenine | C5H5N5 | 135.0545 | 0.6333 | 82 |
|  | 39.0253 | KZ000091 | Pentachlorophenol | C6HCl5O | 263.847 | 0.4295 | 5 |
|  | 39.0253 | OUF00490 | Xanthosine | C10H12N4O6 | 284.07568 | 0.4763 | 44 |
|  | 39.2745 | PR010194 | DL-6,8-Thioctic acid | C8H14O2S2 | 206.04352 | 0.3526 | 34 |
|  | 39.3866 | OUF00262 | Heptadecanoic acid | C17H34O2 | 270.25588 | 0.7254 | 11 |
|  | 39.3866 | OUF00084 | 6-Amino-1-MethylUracil | C5H7N3O2 | 141.05383 | 0.541 | 15 |
|  | 39.4544 | PR010233 | Sebacic acid | C10H18O4 | 202.12051 | 0.359 | 44 |
|  | 39.5387 | OUF00017 | 3-Amino-2-hydroxypyridine | C5H6N2O | 110.04801 | 0.3355 | 12 |
|  | 39.5854 | OUF00084 | 6-Amino-1-Methyl Uracil; | C5H7N3O2 | 141.05383 | 0.5708 | 23 |
|  | 39.6133 | KZ000035 | Hypoxanthine | C5H4N4O | 136.03851 | 0.3126 | 36 |
|  | 40.0409 | OUF00146 | Cyanine | C29H35N2 | 411.28002 | 0.7652 | 12 |
|  | 40.3533 | OUF00482 | UDP-D-Glucuronic acid; | C15H22N2O18P2 | 580.03428 | 0.2184 | 16 |
|  | 40.7373 | OUF00330 | L-Theanine | C12H17NS | 207.10817 | 0.4529 | 4 |
|  | 40.7373 | OUF00383 | Octanoic acid | C8H16O2 | 144.11503 | 0.5289 | 18 |
|  | 40.8277 | PR010237 | Stearic acid | C18H36O2 | 284.27153 | 0.8164 | 71 |
|  | 40.9654 | PR010203 | gamma-Linolenic acid | C18H30O2 | 278.22458 | 0.7711 | 99 |
|  | 41.0625 | OUF00357 | Methyl jasmonic acid | C13H20O3 | 224.14124 | 0.6581 | 76 |
|  | 41.1648 | PR010209 | Oleic acid | C18H34O2 | 282.25588 | 0.399 | 52 |
|  | 41.4006 | OUF00212 | D-Ribulose-5-phosphate | C5H11O8P | 230.01915 | 0.7928 | 11 |
|  | 41.5790 | OUF00416 | 4-Coumaric acid | C9H8O3 | 164.04734 | 0.4362 | 63 |
|  | 41.9487 | KZ000253 | Non-adecanoic acidmethylester | C20H40O2 | 312.30283 | 0.5151 | 40 |
|  | 42.0737 | KZ000175 | 3-Aminopropionitrile | C3H6N2 | 70.0531 | 0.3459 | 8 |
|  | 42.3259 | KZ000011 | DL-6,8-Thioctic acid | C8H14O2S2 | 206.04352 | 0.2608 | 16 |
|  | 42.3515 | KZ000175 | 3-Aminopropionitrile | C3H6N2 | 70.0531 | 0.4634 | 4 |
|  | 42.3515 | OUF00148 | Cytosine | C4H5N3O | 111.04326 | 0.2352 | 14 |
|  | 42.3778 | OUF00184 | DL-3,4-Dihydroxymandelic acid | C8H8O5 | 184.03717 | 0.6854 | 8 |
|  | 42.5435 | OUF00307 | L-Glutathione(oxidizedform) | C20H32N6O12S2 | 612.15196 | 0.7079 | 37 |
|  | 42.5947 | OUF00126 | Terephthalic acid | C8H6O4 | 166.02661 | 0.3718 | 8 |
|  | 42.8973 | KZ000215 | 3-Indoleacetonitrile | C10H8N2 | 156.06875 | 0.3113 | 10 |
|  | 43.1232 | OUF00078 | 5-HydroxymethylUracil | C5H6N2O3 | 142.03784 | 0.3809 | 22 |
|  | 43.2745 | KZ000012 | Adenine; | C5H5N5 | 135.0545 | 0.6537 | 45 |
|  | 43.5019 | OUF00330 | L-Theanine | C12H17NS | 207.10817 | 0.5054 | 18 |
|  | 43.5440 | OUF00274 | Icosanoic acid | C20H40O2 | 312.30283 | 0.5392 | 9 |
|  | 43.5440 | OUF00274 | Icosanoic acid | C20H40O2 | 312.30283 | 0.7501 | 41 |
|  | 44.2963 | OUF00220 | D-Xylulose | C5H10O5 | 150.05282 | 0.5167 | 34 |
|  | 46.9328 | KZ000033 | Guanosine | C10H13N5O5 | 283.09167 | 0.3715 | 65 |
|  | 46.8532 | KZ000152 | L-Cysteine | C3H7NO2S | 121.01975 | 0.5311 | 40 |
|  | 47.1127 | KZ000253 | Nonadecanoic acidmethylester | C20H40O2 | 312.30283 | 0.4372 | 63 |
|  | 47.3521 | PR010147 | Diethanolamine | C4H11NO2 | 105.07898 | 0.4378 | 14 |
|  | 47.3521 | PR010029 | Uridine | C9H12N2O6 | 244.06954 | 0.4696 | 41 |
|  | 47.3754 | OUF00129 | Biopteri | C9H11N5O3 | 237.08619 | 0.2783 | 15 |
|  | 47.5448 | KZ000173 | DOPA | C9H11NO4 | 197.06881 | 0.471 | 21 |
|  | 47.6510 | OUF00124 | Behenic acid | C22H44O2 | 340.33413 | 0.5568 | 58 |
|  | 48.1472 | OUF00099 | Adenylosuccinic acid | C14H18N5O11P | 463.07404 | 0.3095 | 27 |
|  | 48.4205 | OUF00311 | Lignoceric acid | C24H48O2 | 368.36543 | 0.7559 | 29 |
|  | 48.6433 | OUF00223 | (-)-Epicatechin | C15H14O6 | 290.07904 | 0.52 | 35 |
|  | 48.7796 | OUF00015 | 2,6-Diaminopurine | C5H6N6 | 150.06539 | 0.493 | 41 |
|  | 49.1259 | OUF00267 | Homogentisic acid | C8H8O4 | 168.04226 | 0.2513 | 30 |
|  | 49.1598 | OUF00099 | Adenylosuccinic acid | C14H18N5O11P | 463.07404 | 0.3398 | 56 |
|  | 49.1598 | OUF00214 | D-Glucaric acid | C6H10O8 | 210.03757 | 0.2196 | 28 |
|  | 49.1756 | OUF00090 | Acetaminophenglucuronide | C14H17NO8 | 327.09542 | 0.2636 | 29 |
|  | 49.9450 | OUF00216 | D-Sorbitol6-phosphate | C6H15O9P | 262.04537 | 0.1798 | 20 |
|  | 49.9450 | OUF00267 | Homogentisic acid | C8H8O4 | 168.04226 | 0.3479 | 49 |
|  | 50.4773 | OUF00153 | D-(+)-Raffinose | C18H32O16 | 504.16903 | 0.2657 | 15 |
|  | 60.4086 | OUF00088 | 7,8-Dihydroneopterin | C9H13N5O4 | 255.09675 | 0.3439 | 64 |
|  | 50.7574 | OUF00155 | Daidzein | C15H10O4 | 254.05791 | 0.2201 | 13 |
|  | 51.4553 | KZ000203 | D-Glucose 6-phosphate | C6H13O9P | 260.02972 | 0.5293 | 174 |
|  | 51.4862 | OUF00360 | Myricetin | C15H10O8 | 318.03757 | 0.4733 | 142 |
|  | 51.5246 | KZ000183 | alpha-Methyl-DL-serine | C4H9NO3 | 119.05824 | 0.5233 | 241 |
|  | 51.5630 | KZ000203 | D-Glucose 6-phosphate | C6H13O9P | 260.02972 | 0.4976 | 136 |
|  | 51.5916 | OUF00173 | D-Glucosamine 6-phosphate | C6H14NO8P | 259.0457 | 0.8262 | 33 |
|  | 52.0373 | OUF00130 | Biotin | C10H16N2O3S | 244.08816 | 0.5521 | 41 |
|  | 52.3219 | OUF00485 | Uric acid | C5H4N4O3 | 168.02834 | 0.4886 | 57 |
|  | 57.7825 | OUF00469 | Sulfanilamide | C6H8N2O2S | 172.03065 | 0.8039 | 8 |
|  | 59.1272 | OUF00360 | Myricetin | C15H10O8 | 318.03757 | 0.2465 | 51 |
|  | 59.3236 | OUF00088 | 7,8-Dihydroneopterin | C9H13N5O4 | 255.09675 | 0.2785 | 65 |
|  | 59.4389 | OUF00373 | N-Acetylneuraminic acid | C11H19NO9 | 309.10598 | 0.6424 | 13 |
|  | 59.4389 | KZ000144 | Indoleacetic acid | C10H9NO2 | 175.06333 | 0.7179 | 48 |
|  | 59.5450 | KZ000210 | rac-Glycerol 3-phosphoate | C3H9O6P | 172.01367 | 0.4153 | 61 |
|  | 59.5450 | OUF00448 | D-Ribulose 1,5-diphosphate | C5H12O11P2 | 309.98548 | 0.6255 | 116 |
|  | 59.5962 | OUF00323 | L-Saccharopine | C11H20N2O6 | 276.13214 | 0.5962 | 80 |
|  | 62.1539 | OUF00158 | D-Arabinose 5-phosphate | C5H11O8P | 230.01915 | 0.6456 | 5 |

**Supplementary Table 5 Results of Metabolite Set Enrichment Analysis of altered metabolites upon B[a]P exposure**

MESA or Metabolite Set Enrichment Analysis was used to identify biologically meaningful patterns that are significantly enriched in quantitative metabolomic data. The method use Over Representation Analysis (ORA) for the enrichment analysis. ORA is implemented using the hypergeometric test to evaluate whether a particular metabolite set is represented more than expected by chance within the given compound list. One-tailed p values are provided after adjusting for multiple testing. Table 2 below summarizes the result.


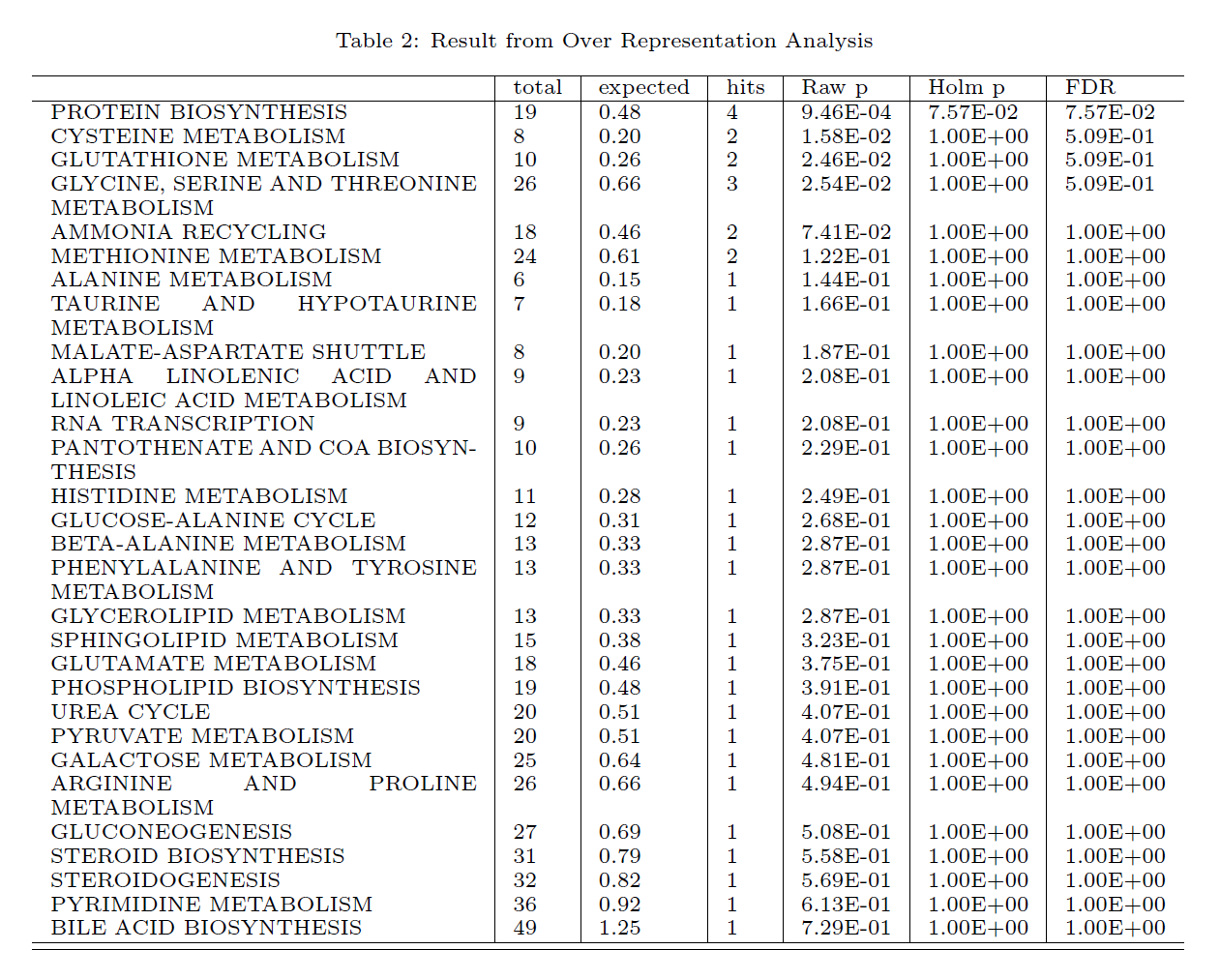

Supplement: Supplementary file 1 — Supplemetary information [file 41598_2017_9936_MOESM1_ESM.doc]
